# A new estimate of afrotherian phylogeny based on simultaneous analysis of genomic, morphological, and fossil evidence

**DOI:** 10.1186/1471-2148-7-224

**Published:** 2007-11-13

**Authors:** Erik R Seiffert

**Affiliations:** 1 Department of Anatomical Sciences, Stony Brook University, Stony Brook, New York, 11794-8081, USA

## Abstract

**Background:**

The placental mammalian clade Afrotheria is now supported by diverse forms of genomic data, but interordinal relationships within, and morphological support for, the group remains elusive. As a means for addressing these outstanding problems, competing hypotheses of afrotherian interordinal relationships were tested through simultaneous parsimony analysis of a large data set (> 4,590 parsimony informative characters) containing genomic data (> 17 kb of nucleotide data, chromosomal associations, and retroposons) and 400 morphological characters scored across 16 extant and 35 extinct afrotherians.

**Results:**

Parsimony analysis of extant taxa alone recovered the interordinal topology (Afrosoricida, ((Macroscelidea, Tubulidentata), (Hyracoidea, (Proboscidea, Sirenia)))). Analysis following addition of extinct taxa instead supported Afroinsectivora (Afrosoricida + Macroscelidea) and Pseudoungulata (Tubulidentata + Paenungulata), as well as Tethytheria (Proboscidea + Sirenia). This latter topology is, however, sensitive to taxon deletion and different placements of the placental root, and numerous alternative interordinal arrangements within Afrotheria could not be statistically rejected. Relationships among extinct stem members of each afrotherian clade were more stable, but one alleged stem macroscelidean (*Herodotius*) never grouped with that clade and instead consistently joined pseudoungulates or paenungulates. When character transformations were optimized onto a less resolved afrotherian tree that reflects uncertainty about the group's interordinal phylogeny, a total of 21 morphological features were identified as possible synapomorphies of crown Afrotheria, 9 of which optimized unambiguously across all character treatments and optimization methods.

**Conclusion:**

Instability in afrotherian interordinal phylogeny presumably reflects rapid divergences during two pulses of cladogenesis – the first in the Late Cretaceous, at and just after the origin of crown Afrotheria, and the second in the early Cenozoic, with the origin of crown Paenungulata. Morphological evidence for divergences during these two pulses either never existed or has largely been "erased" by subsequent evolution along long ordinal branches. There may, nevertheless, be more morphological character support for crown Afrotheria than is currently assumed; the features identified here as possible afrotherian synapomorphies can be further scrutinized through future phylogenetic analyses with broader taxon sampling, as well as recovery of primitive fossil afrotherians from the Afro-Arabian landmass, where the group is likely to have first diversified.

## Background

The monophyly of the supraordinal placental mammalian clade Afrotheria, whose living members include the endemic Afro-Arabian aardvarks (order Tubulidentata), elephant-shrews or sengis (order Macroscelidea), golden moles (family Chrysochloridae), tenrecs (superfamily Tenrecoidea), sea cows (order Sirenia), hyraxes (order Hyracoidea), and elephants (order Proboscidea), is now strongly supported by diverse forms of genomic data, including indels [1,2], SINEs [3,4], "protein sequence signatures" [5], chromosomal syntenies [6], and nuclear and mitochondrial DNA sequences [2]. Although Afrotheria is estimated to have had the longest stem lineage (about 25 Myr) of all extant placental supraordinal clades [[7], but see [8]], morphological support for afrotherian monophyly is elusive [9-11]. A few morphological features have been mapped onto molecular phylogenies as synapomorphies of crown Afrotheria [12-15], but morphological phylogenetic analyses of the placental mammal radiation continue to favor afrotherian polyphyly [e.g., [16,17]].

An important first step in addressing the problem of morphological character support for Afrotheria will be resolution of the phylogenetic position of tenrecs and golden moles (order Afrosoricida) within that clade [18,19]. Afrosoricids stand apart from sengis, aardvarks, and paenungulates (hyracoids, sirenians, and proboscideans) in that the latter are all thought to be derived from "proto-ungulate" or "condylarth" ancestors [20-24], whereas afrosoricids – which were formerly placed in the order Lipotyphla alongside hedgehogs, shrews, moles, and solenodons (now Eulipotyphla) – share a number of seemingly primitive morphological features with eulipotyphlans and Cretaceous stem placentals. Phylogenetic analyses of the longest available concatenation of afrotherian DNA sequences [2] nevertheless nest tenrecs and golden moles deep within Afrotheria, with Macroscelidea and Tubulidentata placed as consecutive sister taxa to Afrosoricida within a clade that has been named Afroinsectiphillia [25]. The monophyly of Afroinsectiphillia, but not Afroinsectivora, is also supported by a single SINE [4] and a unique chromosomal synteny [6], while Afroinsectiphillia and Afroinsectivora, but not Afrosoricida, are supported by a recent analysis of LINE-1 [26]. This phylogenetic pattern implies that the "proto-ungulate" features shared by sengis, aardvarks, and paenungulates might have evolved along the afrotherian stem lineage, and that afrosoricid morphology represents a remarkable case of taxic atavism [18,27].

Another outstanding problem in afrotherian phylogenetics is the branching order among Hyracoidea, Proboscidea and Sirenia within Paenungulata. Various types of genomic data have been collected in an effort to resolve paenungulate relationships [2,4,28,29], but this information has consistently given either weak or contradictory signals, ultimately leaving researchers with a seemingly unresolvable trichotomy [30]. These results contrast with morphological evidence, which most clearly supports a sirenian-proboscidean clade (Tethytheria) within Paenungulata [16,31-33]. Among other things, the monophyly versus paraphyly of Tethytheria could have important implications for our understanding of the adaptations of the ancestral crown paenungulate, because early fossil proboscideans and sirenians are generally found in near-shore or marine deposits [34,35] that suggest an early preference for semi-aquatic habitus. If Tethytheria is monophyletic, this adaptive pattern is best explained as having been due to common ancestry, whereas if the group is paraphyletic, semi-aquatic habitus either evolved convergently in early proboscideans and sirenians, or was an ancestral feature of Paenungulata as a whole.

The extant members of afrotherian orders differ dramatically in their morphology and adaptations, and represent the tips of long branches that extend well back into the Paleocene and/or Late Cretaceous [7]. Extinct taxa should play a critical role in efforts to resolve placental supraordinal phylogeny because fossils exhibit unique combinations of primitive and derived characters that help to break up long branches [36] that otherwise might attract due to homoplasy rather than homology. The only recent phylogenetic analysis to have scored members of all extant afrotherian orders included only two undoubted fossil afrotherians, however, both of which were extinct paenungulates [16]. Furthermore, a recent phylogenetic analysis that included more fossil afrotherians [37], and which recovered a macroscelidean-paenungulate clade to the exclusion of perissodactyls and artiodactyls, did not sample aardvarks, tenrecs and golden moles, which lack some or all of the features that support the macroscelidean-paenungulate clade recovered in that study.

This study includes 400 morphological characters scored across 16 extant and 35 extinct afrotherians, and is combined with chromosomal associations [6], retroposons [4], and > 17 kb of nucleotide data [2] to create the single largest phylogenetic data set (at least 4,590 parsimony informative characters) that has yet been brought to bear on the interrelationships of living and extinct afrotherians. Included in the morphological partition are new data on recently published afrotherian fossil material from the Paleogene of north Africa [34,38,39] as well as undescribed late Eocene hyracoids, macroscelideans, and afrosoricids from Egypt. Parsimony analysis of these data reveals a new hypothesis of relationships within Afrotheria, and highlights a central role for Paleogene "elephant-shrews" in afrotherian phylogenetics.

## Results

### Phylogenetic analysis of extant taxa alone

**Figure 1:**
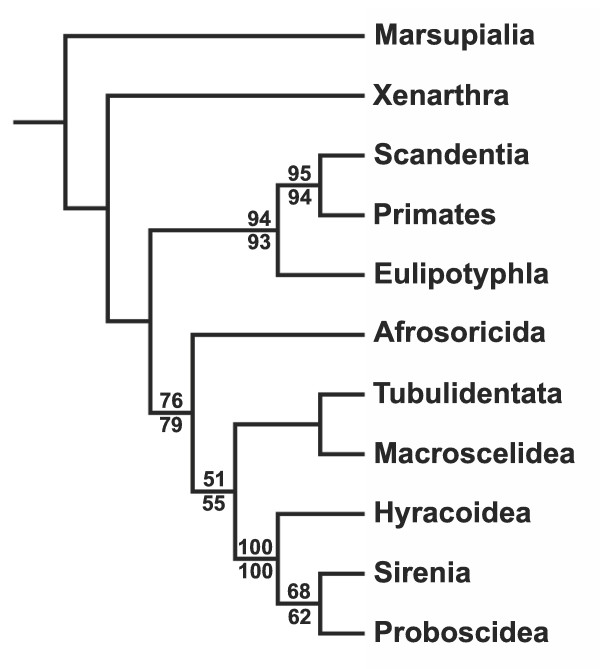
**Estimate of afrotherian interordinal phylogeny based on data from extant taxa alone**. Strict consensus of results from parsimony analyses of extant taxa only with all characters unordered (1 most parsimonious tree (MPT), tree length (TL) = 18428, consistency index (CI) = 0.52, retention index (RI) = 0.39, rescaled consistency index (RCI) = 0.26) and with some multistate characters ordered and scaled (1 MPT, TL = 18068, CI = 0.52, RI = 0.39, RCI = 0.26). Intraordinal relationships are not shown, but in both trees are as in Fig. 2. Numbers above and below branches are bootstrap support values (1000 replicates) from analysis of the matrix with some multistate characters ordered and scaled (above) and with all multistate characters unordered (below).

Regardless of how morphological characters were treated [i.e., with selected multistate characters either unordered (= UA, unordered analysis), or ordered and scaled (= OSA, ordered and scaled analysis)], simultaneous analysis of extant taxa alone recovered Paenungulata, Tethytheria, a Macroscelidea-Tubulidentata clade, and a Macroscelidea-Tubulidentata-Paenungulata clade to the exclusion of Afrosoricida (Fig. 1). Aside from monophyly of Paenungulata, these results are at odds with the relationships recovered by Amrine-Madsen et al. [2], although among these supraordinal clades only Paenungulata had high bootstrap support (Fig. 1). With Afrosoricida placed as the sister group of all other afrotherians, there was only one unambiguously optimized morphological synapomorphy of Afrotheria in the OSA (placement of the root of the zygomatic lateral to M3), none in the UA, but 24 (UA) to 30 (OSA) ambiguous morphological synapomorphies of that clade. The Macroscelidea-Tubulidentata-Paenungulata clade was supported by 69 (OSA) to 71 (UA) ambiguously and 22 (UA) to 26 (OSA) unambiguously optimized morphological synapomorphies.

**Figure 2:**
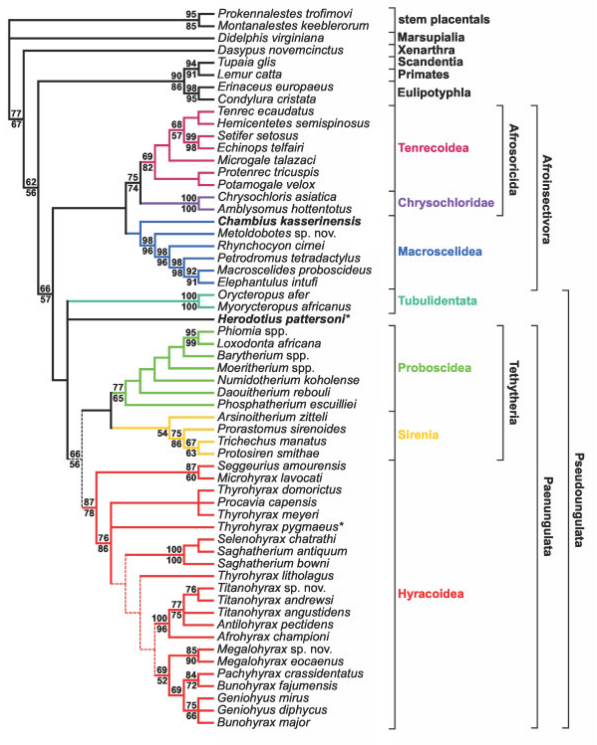
**Phylogenetic relationships of living and extinct afrotherians**. Adams consensus tree summarizing results from parsimony analyses with all characters unordered (12 MPTs, TL = 19478, CI = 0.50, RI = 0.44, RCI = 0.28) and with some morphological characters ordered and scaled (1 MPT, TL = 18689.54, CI = 0.50, RI = 0.44, RCI = 0.28). Branches depicted with dashes break down in the strict consensus of all 13 trees. Values above and below branches are bootstrap support (1000 replicates) from analysis of the matrix with some multistate characters ordered and scaled (above) and with all multistate characters unordered (below). Herodotiine taxa (alleged stem macroscelideans) are in bold face; asterisks identify "wild card" taxa whose variable positions given different character treatments lead to decreased resolution in the strict consensus tree.

### Phylogenetic analysis following addition of extinct taxa

Parsimony analysis following addition of 35 extinct afrotherian species recovered a supraordinal branching pattern that is more consistent with Amrine-Madsen et al.'s [2] tree based solely on molecular data. The Macroscelidea-Tubulidentata clade recovered in the analysis of extant taxa alone breaks down, and Macroscelidea joined Afrosoricida, forming a weakly supported Afroinsectivora. The primary differences from Amrine-Madsen et al.'s [2] tree are the placement of aardvarks with Paenungulata rather than Afroinsectivora, forming "Pseudoungulata" [40], and of Hyracoidea as the sister group of Tethytheria rather than of Proboscidea alone. Outside of Paenungulata, the branching order among afrotherians was the same as in Amrine-Madsen et al.'s [2] tree, but the root was placed between Afroinsectivora and Pseudoungulata rather than between Afroinsectiphillia and Paenungulata. Inclusion of fossil taxa led to reduced bootstrap support for both Paenungulata and Tethytheria, in the former case due in part to the variable placement of the alleged stem macroscelidean *Herodotius*, which in different equally parsimonious trees emerged as the sister taxon of either Pseudoungulata, Tubulidentata, Paenungulata, or Hyracoidea, but never as a sister group of crown Macroscelidea. The lower support for Tethytheria can be explained by the inclusion of primitive fossil proboscideans and sirenians, which reveal that a number of the apomorphies that were unambiguously optimized as tethytherian synapomorphies in the analysis of extant taxa alone are in fact more parsimoniously explained as homoplasies rather than homologies [e.g., [34,41]].

Although it is interesting that the addition of fossil taxa led to an improved fit with the tree derived from maximum likelihood and Bayesian analysis of the molecular data alone, closer examination reveals that this most parsimonious topology is not particularly stable. For instance, trees derived from analyses that were constrained to recover the interordinal arrangement (Afrosoricida, (Paenungulata, (Macroscelidea-Tubulidentata))) were only two steps longer than the unconstrained tree, and could not be statistically rejected; nor could the alternative arrangement of a monophyletic Afroinsectiphillia containing Afroinsectivora (Table 1). None of the three possible arrangements of the paenungulate orders were either well supported or rejected by statistical tests, and even afrosoricid diphyly (e.g., with either Tenrecoidea or Chrysochloridae placed as the sister taxon of Macroscelidea) could not be rejected (Table 1). Furthermore, if the well-known Oligocene embrithopod *Arsinoitherium *was excluded from the analysis, Afrosoricida was again placed as the sister group of a (Paenungulata, (Macroscelidea, Tubulidentata)) clade, within which Hyracoidea was placed as the sister taxon of Proboscidea to the exclusion of Sirenia (Table 2). Such a major impact of a single taxon such as *Arsinoitherium *is unsettling, given that it is a highly autapomorphic late-surviving genus that evolved a number of morphological convergences with proboscideans [34] – some of which could be optimized as tethytherian synapomorphies given *Arsinoitherium*'s placement as a stem sirenian in the present analysis. Deletion of basal Eocene taxa, such as the herodotiines *Chambius *and *Herodotius*, the hyracoids *Microhyrax *and *Seggeurius*, the proboscidean *Phosphatherium*, and the sirenian *Prorastomus *did not alter the optimal interordinal topology (Table 2), but afrotherian interordinal phylogeny did prove to be sensitive to placement of the root of the placental tree: if Exafroplacentalia (Xenarthra + Boreoeutheria) was constrained to be monophyletic, Afroinsectivora again broke down and Macroscelidea was placed as the sister taxon of other afrotherians (Table 2). Interestingly, when the monophyly of Atlantogenata (Xenarthra + Afrotheria) was constrained, Afroinsectiphillia, Afroinsectivora, and a hyracoid-sirenian clade were recovered in the OSA (Table 2).

**Table 1:** Tests of alternative interordinal hypotheses within Afrotheria

		Number of trees	Assumption set	Tree length	Consistency index	Retention index	Rescaled consistency index	Macroscelidea-Tubulidentata?	Afroinsectivora?	Tethytheria?	Hyracoidea-Proboscidea?	Hyracoidea-Sirenia?	Templeton test (P)
	
	Optimal topology	1	OS	18689.5	0.50	0.44	0.28	No	Yes	Yes	No	No	-
	
		12	U	19478	0.50	0.44	0.28	No	Yes	Yes	No	No	-
Alternative hypothesis	Afroinsectiphillia	1	OS	18695	0.50	0.44	0.28	No	Yes	No	No	Yes	0.838
		12	U	19484	0.49	0.44	0.28	No	Yes	No	No	Yes	0.738
	Macroscelidea + Tubulidentata	1	OS	18691.7	0.50	0.44	0.28	NA	NA	Yes	No	No	0.722
		6	U	19480	0.50	0.44	0.28	NA	NA	Yes	No	No	0.900
	Afrosoricida + Tubulidentata	1	OS	18702.5	0.50	0.44	0.28	NA	NA	Yes	No	No	0.284
		3	U	19490	0.49	0.44	0.28	NA	NA	Yes	No	No	0.440
	Macroscelidea + Tenrecoidea	1	OS	18712.6	0.50	0.44	0.28	NA	Yes	No	No	Yes	0.415
		43	U	19506	0.49	0.44	0.28	NA	Yes	**No**	No	**Yes**	0.194
	Chrysochloridae + Macroscelidea	1	OS	18701.2	0.49	0.44	0.28	NA	No	Yes	No	No	0.742
		120	U	19503	0.49	0.44	0.28	NA	No	Yes	No	No	0.634
	Herodotiinae + Macroscelidea	1	OS	18692.3	0.50	0.44	0.28	No	Yes	Yes	No	No	0.432
		27	U	19481	0.49	0.44	0.28	No	No	Yes	No	No	0.702
	Hyracoidea + Sirenia	1	OS	18693.6	0.50	0.44	0.28	No	Yes	NA	NA	NA	0.835
		18	U	19481	0.49	0.44	0.28	Yes	No	NA	NA	NA	0.885
	Hyracoidea + Proboscidea	1	OS	18693	0.50	0.44	0.28	Yes	No	NA	NA	NA	0.797
		74	U	19483	0.49	0.44	0.28	**Yes**	No	NA	NA	NA	0.810

Statistics for most parsimonious trees derived from analyses that were constrained to agree with alternative phylogenetic hypotheses. Consistency index excludes uninformative characters. Value for Templeton test (P, calculated in PAUP 4.0b10) is the probability of finding a more extreme T-value under the null hypothesis of no difference between the two trees (two-tailed test), arbitrarily calculated by comparing the first tree in each of the two alternative tree lists. Results in bold are those that are present in the Adams consensus of all equally parsimonious trees. OS = ordered and scaled analysis; U = unordered analysis; NA = not applicable.

**Table 2:** Sensitivity of afrotherian interordinal phylogeny to alternative placements of the placental root, taxon deletion, and taxon addition

**Hypothesis**		**Afrotherian interordinal phylogeny**	
Exafroplacentalia	OS	(Macroscelidea, (Afrosoricida, (Tubulidentata, (Proboscidea, (Hyracoidea, Sirenia)))))	
	U	(Macroscelidea, (Afrosoricida, (Tubulidentata, (Hyracoidea, (Proboscidea, Sirenia)))))*	
Atlantogenata	OS	((Tubulidentata, (Macroscelidea, Afrosoricida)), (Proboscidea, (Hyracoidea, Sirenia)))	
	U	no change	
**Deleted taxon**			
*Arsinoitherium*	OS	(Afrosoricida, ((Macroscelidea, Tubulidentata), (Sirenia, (Hyracoidea, Proboscidea))))	
	U	(Afrosoricida, ((Macroscelidea, Tubulidentata), (Sirenia, (Hyracoidea, Proboscidea))))	
*Chambius*	OS	no change	
	U	no change	
*Herodotius*	OS	no change	
	U	no change*	
*Microhyrax*	OS	no change	
	U	no change	
*Phosphatherium*	OS	no change	
	U	no change	
*Prorastomus*	OS	no change	
	U	no change*	
*Seggeurius*	OS	no change	
	U	no change	
**Added taxon**		**Sister taxon**	**Afrotherian interordinal phylogeny**
*Kelba*	OS	Tubulidentata	no change
	U	Tubulidentata	no change
*Widanelfarasia*	OS	within Tenrecoidea	no change
	U	within Tenrecoidea	no change
*Kelba *+			
*Widanelfarasia*	OS	no change	no change
	U	no change	no change

Interordinal relationships within Afrotheria from (above) analyses constrained to agree with the alternative hypotheses Exafroplacentalia (Afrotheria, (Boreoeutheria, Xenarthra)) and Atlantogenata (Boreoeutheria, (Afrotheria, Xenarthra)), and (below) analyses run following deletion or addition of various extinct taxa. Results followed by an asterisk are those that occur in the Adams consensus of all equally parsimonious trees (the strict consensus of which was less resolved than the optimal topologies based on analyses including all taxa). OS = ordered and scaled analysis; U = unordered analysis.

### Relationships among fossil taxa

The phylogeny of the diverse Paleogene paenungulate radiation has never been analyzed within the context of afrotherian monophyly. One of the novel results of this analysis is the placement of enigmatic *Arsinoitherium *as a stem sirenian rather than as a stem proboscidean [16,42,43] or stem tethytherian [34]. Within Proboscidea, most debate revolves around the placement of middle-late Eocene *Moeritherium*, which is variously seen as a late-surviving basal form or as more deeply nested within the proboscidean radiation, sharing more recent common ancestry with Oligocene-Recent elephantiforms than with older taxa [44,45]. The results of the current analysis are more consistent with the stratigraphic succession of early proboscideans in placing the oldest taxa (earliest Eocene *Phosphatherium *and *Daouitherium *[34,46,47]) as the most basal forms, slightly younger *Numidotherium koholense *[48] as the sister group of middle Eocene-to-Recent proboscideans, and *Moeritherium *in a more nested position. *Moeritherium*'s enigmatic contemporary *Barytherium*, which was recently placed with *Daouitherium *in a more restricted analysis [34], was placed as the sister group of elephantiforms to the exclusion of all other Eocene taxa.

The hyracoid phylogeny recovered in this analysis is very different from all previous estimates, none of which took into account multiple afrotherian outgroups [49-51]. The results are again consistent with stratigraphic succession in placing the oldest (early and early middle Eocene, respectively) taxa *Seggeurius *[52] and *Microhyrax *[37,51] as the sister group of all younger hyracoids. In contrast to previous studies that positioned *Geniohyus *as the sister taxon of other hyracoid genera [51,53], in this study the small-bodied Paleogene forms *Thyrohyrax *and *Saghatherium *are consecutive sister taxa of large-bodied forms including *Geniohyus*. The genus *Thyrohyrax *was consistently found to be polyphyletic, with only the type species (*Thyrohyrax domorictus*) and *Thyrohyrax meyeri *forming a clade along with extant *Procavia*. *Geniohyus *was found to be nested deep within the Paleogene radiation in a clade containing species of *Bunohyrax *and *Pachyhyrax*. A clade containing *Antilohyrax*, *Titanohyrax*, and early Miocene *Afrohyrax *was also well-supported, and placed as the sister group of a *Bunohyrax-Geniohyus-Megalohyrax-Pachyhyrax *clade.

The fossil record of non-paenungulate afrotherians is relatively poor, although the Eocene herodotiines *Chambius *and *Herodotius *are generally considered to be primitive macroscelideans [20,21,37]. Undescribed new material of late Eocene *Metoldobotes*, including cranial remains, helps to place that genus as the sister taxon of crown Macroscelidea with strong bootstrap support (Fig. 2), but *Herodotius *was consistently placed alongside "pseudoungulates" rather than macroscelideans (see discussion below). A placement of both herodotiines as stem macroscelideans could not be statistically rejected, however (Table 1). Within Tenrecoidea, early Miocene *Protenrec *was placed as a sister genus of extant *Potamogale*, suggesting that differentiation of crown tenrecoids might have already occurred by that time [54]. Addition of two enigmatic African fossil placentals – early Miocene *Kelba *and late Eocene *Widanelfarasia *– does not alter the scheme of interordinal relationships supported by the full taxon set. Regardless of how characters are treated, *Kelba *is placed as a stem member of Tubulidentata, lending support to a previous suggestion that ptolemaiids might be aligned with aardvarks [55], while *Widanelfarasia *nests within crown Tenrecoidea as the sister taxon of *Protenrec *(Table 2).

### Morphological character support for Afrotheria

Despite the placement of afrosoricids with macroscelideans in the analysis of living and extinct taxa, under this arrangement afrotherian monophyly is not unambiguously supported by any of the "proto-ungulate" features that macroscelideans share with aardvarks and paenungulates. In fact the existence of any unambiguous morphological character support for Afrotheria given this tree is dependent on whether delayed or accelerated optimization is used; under delayed transformation, there are four unambiguous synapomorphies of Afrotheria regardless of how multistate characters are treated (presence of a naviculocalcaneal facet, scattered vomeronasal organ blood vessels [13], placement of the internal carotid lateral to the anterior pole of pars cochlearis [13], and four allantoic vessel chambers [15]). An additional four features [presence of a small P3 protocone, presence of well-developed buccal cingula rather than stylar shelves, increase in lumbar vertebra number from 6 to 8, and testicondy (intrabdominal testes)] emerge as unambiguous synapomorphies depending on treatment of certain multistate characters. Under accelerated transformation, there are no unambiguous afrotherian synapomorphies, but there are 31 (OSA) and 33 (UA) ambiguous synapomorphies of that clade.

Given that there can be little confidence in any of the proposed arrangements of Afrosoricida, Macroscelidea, or Tubulidentata within Afrotheria, an alternative, and perhaps more conservative, approach is to optimize characters onto an afrotherian phylogeny that is less resolved at the supraordinal level. With Macroscelidea, Tubulidentata, Afrosoricida, and *Herodotius *forming a basal polytomy within Afrotheria, *Kelba *placed as a stem member of Tubulidentata, *Widanelfarasia *nested within crown Tenrecoidea, and with relationships among Hyracoidea, Sirenia, and Proboscidea unresolved, a total of 21 morphological features are identified as unambiguous afrotherian synapomorphies across the different assumption sets (Table 3). Of these characters, nine are congruently optimized as afrotherian synapomorphies regardless of how transformations are optimized (accelerated or delayed) or how multistate characters are treated (ordered and scaled or unordered).

**Table 3:** Morphological character support for Afrotheria

**Character number and state change**	**Description of character state transformation**
35 (0=>3 **or **0=>2)	p4 paraconid small ==> p4 paraconid large and distinct (OSA_AT, UA_AT, UA_DT) **or **p4 paraconid small ==> p4 paraconid variably large and distinct (OSA_DT)
36 (1=>2)	p4 metaconid present but small relative to the protoconid ==> p4 metaconid present and approximately as large as the protoconid (OSA_DT, UA_DT)
37 (0=>2)	p4 protolophid absent ==> p4 protolophid incipient (OSA_DT)
39 (0=>2 **or **0=>1)	p4 entoconid absent ==> p4 entoconid present, smaller than hypoconid (OSA_AT) **or **p4 entoconid absent ==> p4 entoconid variably present, smaller than hypoconid (OSA_DT)
40 (1=>2)	p4 hypoconid less than half the height of protoconid ==> p4 hypoconid large, greater than half the height of the protoconid (OSA_AT, OSA_DT, UA_AT, UA_DT)
41 (0=>1)	p4 hypolophid absent ==> incipient p4 hypolophid variably present (OSA_DT)
45 (0=>2)	p4 talonid narrower than trigonid ==> p4 talonid equal in width to the trigonid (OSA_AT, OSA_DT)
73 (0=>2 **or **0=>1)	Cristid obliqua on lower molars meets hypocristid at a sharp angle ==> junction between cristid obliqua and hypocristid more open, buccal aspect of hypoconid rounded (OSA_AT, UA_AT, UA_DT) **or **cristid obliqua on lower molars meets hypocristid at a sharp angle ==> junction between cristid obliqua and hypocristid variably more open, buccal aspect of hypoconid rounded (OSA_DT)
77 (2=>1)	Lower molar entocristids present ==> lower molar entocristids variably absent (OSA_AT, OSA_DT)
115 (0=>2)	P3 protocone absent or highly reduced ==> P3 protocone present and small (OSA_AT, OSA_DT, UA_AT, UA_DT)
129 (0=>4)	P4 metacone absent ==> P4 metacone present, distinct, and differentiated from the paracone (OSA_AT, OSA_DT, UA_AT, UA_DT)
143 (0=>4)	M1-2 mesial cingulum broken or absent ==> M1-2 mesial cingulum complete and well-defined across all or most of the mesial face of the teeth (OSA_DT, UA_DT)
150 (0=>4)	Buccolingually extensive shelf present on buccal aspect of upper molars ==> distinct buccal cingulum on upper molars (UA_AT, UA_DT)
165 (2=>0)	M1-2 parastyles small ==> M1-2 parastyles absent (UA_AT, UA_DT)
184 (6=>A)	6 lumbar vertebrae ==> 8 lumbar vertebrae (UA_AT, UA_DT)
218 (0=>4)	Lunar-unciform contact present ==> lunar-unciform contact absent (OSA_DT, UA_DT)
267 (0=>1)	naviculocalcaneal facet absent ==> naviculocalcaneal facet present (OSA_DT, UA_DT)
291 (0=>1)	Vomeronasal organ blood vessels prominent ==> vomeronasal organ blood vessels scattered (OSA_AT, OSA_DT, UA_AT, UA_DT)
383 (0=>1)	Internal carotid medial to anterior pole of pars cochlearis ==> internal carotid lateral to anterior pole of pars cochlearis (OSA_AT, OSA_DT, UA_AT, UA_DT)
388 (5=>0 **or **3=>2 **or **1=>0)	Testes descend into a pendulous scrotum ==> testes intraabdominal and situated near kidneys (UA_AT, UA_DT) **or **testes pass into a cremasteric sac ==> testes migrate to or just through the ventral abdominal wall (OSA_AT) **or **testes migrate to near the bladder ==> testes intraabdominal and situated near kidneys (OSA_DT)
397 (0=>1)	One allantoic vesicle chamber ==> four allantoic vesicle chambers (OSA_AT, OSA_DT, UA_AT, UA_DT)

Character state changes identified as unambiguous morphological synapomorphies of Afrotheria when relationships among Afrosoricida, Macroscelidea, Paenungulata, and Tubulidentata are depicted as unresolved. OSA_AT = ordered and scaled analysis, accelerated transformation; OSA_DT = ordered and scaled analysis, delayed transformation; UA_AT = unordered analysis, accelerated transformation; UA_DT = unordered analysis, delayed transformation.

## Discussion

The instability of afrotherian interordinal relationships is remarkable given that all of the analyses performed here included at least 4,590 parsimony informative molecular and morphological characters. Molecular divergence estimates clearly indicate that cladogenesis among the stem lineages of Tubulidentata, Macroscelidea, Afrosoricida, and Paenungulata occurred rapidly, and probably in the latest Cretaceous; according to the recent estimates provided by Murphy et al. [56], these clades had all diverged within the first 5 million years of crown afrotherian evolution. Morphological evidence for these supraordinal divergences either did not accumulate along these short internal branches, or was subsequently "erased" by evolution along the much longer branches leading to ordinal crown clades.

Considerable ambiguity is introduced by missing data and different methods for optimizing character states onto slightly different afrotherian phylogenies, but it remains distinctly possible that there are a number of morphological synapomorphies of crown Afrotheria, and that the ancestral crown afrotherian more closely resembled a "proto-ungulate" than an "insectivore". For instance, some of the only character transformations that consistently optimized unambiguously onto the afrotherian stem on the less resolved tree (Table 3) are related to molarization of the premolars, which is seen in paenungulates, early macroscelideans, herodotiines, and *Kelba*. With characters unordered, the ancestral afrotherian is also reconstructed as having no parastyles and well-developed buccal cingula rather than stylar shelves, which again suggests that afrosoricids (which do have parastyles and stylar shelves) have undergone reversals to the dental character states observable in more primitive Cretaceous placentals.

Of interest in this regard is the phylogenetic placement of the alleged stem macroscelidean *Herodotius*, from the late Eocene of Egypt, which consistently groups with paenungulates or pseudoungulates to the exclusion of afroinsectivorans, while an older and very similar herodotiine (*Chambius*) is placed in Afroinsectivora with macroscelideans. Herodotiine diphyly is probably an artifact of missing data, but the distribution of herodotiines on both sides of the afrotherian tree again lends some support to the idea that the paenungulate-like dental morphology of herodotiines may be primitive within Afrotheria. The most likely explanation for herodotiine diphyly is that, in known parts, *Chambius *and *Herodotius *overlap solely in having very similar upper and lower molars and fourth premolars, but *Chambius *is now known to have macroscelidean-like astragalar and calcaneal morphology [38], whereas *Herodotius *has somewhat paenungulate-like anterior premolars and incisors (personal observation). If some uniformity of herodotiine morphology is assumed and *Chambius *and *Herodotius *are assigned the character states of the herodotiine taxon that preserves those parts, then these taxa together join Tubulidentata (OSA) or Pseudoungulata (UA Adams consensus), but never Macroscelidea, in parsimony analyses of the data set presented here. Additional cranial or postcranial morphology of herodotiines should play a key role in future efforts to tease apart homology and homoplasy among early afrotherians: if herodotiines are in fact stem macroscelideans within Afroinsectivora, then their detailed dental resemblances to paenungulates will either not be present in older and more primitive stem macroscelideans, or these features will emerge as plesiomorphic within Afroinsectivora and Afrotheria and will support a proto-ungulate origin for both clades.

Of the remaining morphological features that support afrotherian monophyly on the less resolved tree, none clearly point to either a "proto-ungulate" or "insectivore" origin for the clade. Under delayed transformation, at least one morphological feature that was previously thought to be a synapomorphy of Paenungulata (loss of lunar-unciform contact) [57] instead appears as a synapomorphy of Afrotheria as a whole. Other features that have already been identified as probable afrotherian synapomorphies, such as increased lumbar vertebral number [14], cranial soft tissue features [13], testicondy [12], and aspects of placentation [15] also optimize unambiguously as afrotherian synapomorphies across all or most assumption sets. Presence of a contact between the navicular and calcaneus, which occurs in proboscideans, *Arsinoitherium*, and aardvarks as well as some macroscelideans, tenrecs, golden moles, and fossil hyracoids, is here identified for the first time as another possible morphological synapomorphy of crown Afrotheria.

There are a few obvious deficiencies of the present study, some of which should be improved upon in future analyses. One obvious improvement that can be made is greater taxon sampling within Placentalia (and Mammalia more broadly), including a greater diversity of Cretaceous mammals, as all such taxa should help to clarify ancestral character states for crown Placentalia. One obvious criticism of the equally-weighted total evidence approach taken here is that "rare genomic changes" (RGCs) such as retroposons and chromosomal syntenies have been given equal weight to point mutations in DNA sequences, the latter of which are surely much more prone to homoplasy [19]. Unfortunately there is no clear solution to this practical problem aside from arbitrary weighting of RGCs – which, in the absence of a strong theoretical framework for predicting the relative likelihood of, for instance, chromosomal rearrangements relative to point mutations, is here considered to be an untenable approach. The same criticism can certainly also be raised regarding delimitation and treatment of morphological characters (many of which may be of low phylogenetic utility), however the same problem holds [58]. Finally, the use of parsimony rather than likelihood in the analysis of molecular and/or morphological data is arguably not entirely satisfactory and ideally will be addressed in future analyses using programs that allow for mixed models [59]; thus far, however, Bayesian analyses of the current data set have failed to achieve convergence despite considerable computational effort, presumably due in large part to the numerous genomic and morphological characters missing in fossil taxa.

## Conclusion

Simultaneous analysis of ≤ 4,590 parsimony informative genomic and morphological characters scored across 16 extant and 35 extinct afrotherians recovers a monophyletic Afroinsectivora (Afrosoricida + Macroscelidea), Pseudoungulata (Tubulidentata + Paenungulata), and Tethytheria (Proboscidea + Sirenia) within Paenungulata. None of these supraordinal clades are well-supported, however, and phylogenetic alternatives such as Afroinsectiphillia, a (Paenungulata, (Macroscelidea, Tubulidentata)) clade, an Afrosoricida-Tubulidentata clade, afrosoricid diphyly, a Hyracoidea-Proboscidea clade, and a Hyracoidea-Sirenia could not be statistically rejected.

Divergences among Afrosoricida, Macroscelidea, Paenungulata, and Tubulidentata must have occurred very rapidly in the Late Cretaceous, and unambiguous morphological evidence for afrotherian supraordinal clades aside from Paenungulata either does not exist or has been overwritten by subsequent evolution through the Cenozoic. On the optimal topologies derived from these analyses, identification of unambiguous morphological support for afrotherian monophyly is dependent on optimization method, but on a less resolved interordinal phylogeny a total of 21 unambiguous afrotherian synapomorphies are identified, 9 of which appear as such across all character treatments and optimization methods.

Relationships among early fossil members of each afrotherian order are generally consistent with stratigraphic succession; novel results are the placement of *Arsinoitherium *as a stem sirenian, and the placement of the alleged macroscelidean *Herodotius *with pseudoungulates. Additional cranial and postcranial material of Eocene *Chambius*, *Herodotius*, and other enigmatic early African taxa will be of great importance for understanding afrotherian interordinal phylogeny, and will help to test competing hypotheses of a "proto-ungulate" versus "insectivore" origin for that clade. Of utmost importance, however, is the recovery of even earlier fossil afrotherians from previously unsampled early Paleocene and Late Cretaceous horizons in Afro-Arabia.

## Methods

### Morphological data

The 400-character morphological partition draws heavily on previous phylogenetic investigations of afrotherian orders [10,13,42-44,60-63]. Characters provided in these sources were included so long as they were phylogenetically informative given the taxon sample, and could be scored consistently across the range of taxa considered herein. In many cases the morphologically diverse taxon sample required that other authors' definitions of individual character states be modified to maintain phylogenetic information. Other characters in the morphological data set were either discovered while making observations on original material, or were discussed in previously published studies by authors who did not undertake phylogenetic analyses. Taxa expressing polymorphisms were assigned an alternative character state rather than a standard polymorphic coding, as evidence from simulations indicates that the former method may increase phylogenetic accuracy relative to the latter [64]. Ordered multistate characters were scaled so that those characters with multiple alternative "polymorphic" states would not have a disproportionate effect on phylogeny estimation. Myrmecophagous taxa with highly modified teeth (e.g., aardvark and armadillo) were scored as "missing" for dental cusp and crest characters. See Additional file 1 for additional information on character descriptions, methods, and sources of character data.

### Genomic data

The nucleotide partition employed in this analysis is essentially that of Amrine-Madsen et al. [2], combining the > 16.4 kb data set of Murphy et al. [65] with the more recently published afrotherian sequences from the nuclear apolipoprotein B locus [2]. As noted by Madsen et al. [1] and Murphy et al. [66], the concatenations that were combined to create the Murphy et al. [65] data set were aligned using CLUSTAL [e.g., [67]], and in some cases modified following manual inspection. Different sequence alignment and gap treatment options exist [68-70], but no attempt was made to modify Murphy et al.'s [65] published alignment. Following Scally et al. [71], individual gap characters have been scored as missing rather than as an alternative (fifth) character state because indels that are two or more nucleotides in length are likely to be the result of a single event. Following Murphy et al. [65], regions of ambiguous alignment (designated as character set "ambiguous" in their data set) were excluded from analysis. In some cases molecular sequences are chimeric in that data from more than one genus have been combined to create a single intraordinal concatenation. Data on chromosomal associations derive from various sources [6,28,72] and retroposon data are from Nishihara et al. [4]; some non-afrotherian outgroup species are assumed to share the chromosomal association or retroposon presence/absence state of the closest ordinal relative for which such data are available. Although an enormous amount of genomic information has recently been gathered in an attempt to resolve the interrelationships of the clades Afrotheria, Xenarthra, and Boreoeutheria, there is still no agreement on the early branching pattern within Placentalia [56,73,74]. Rather than incorporating these different forms of data into the current analysis, the impact of the competing Atlantogenata, Exafroplacentalia, and Epitheria hypotheses on afrotherian interrelationships was tested simply by constraining the included xenarthran (*Dasypus*) to join Afrotheria or Boreoeutheria in secondary analyses.

Character data were compiled in the program Nexus Data Editor [75]. See additional file 2 for the complete data set. Parsimony analyses were performed in PAUP 4.0 b10 [76] using heuristic searches, random addition sequence, and the tree bisection-reconnection branch swapping algorithm across 1000 replicates. Comparisons of alternative topologies to the optimal topology were also performed in PAUP 4.0 b10 using Templeton (Wilcoxon signed-rank) tests. All consistency indices reported herein were calculated with uninformative characters excluded.

### Taxon sampling

The taxon set includes extant representatives of all afrotherian orders and samples much of the morphological diversity observable within ordinal crown clades. As one of the primary goals of this study was to break up the "long branches" that separate autapomorphic extant taxa from supraordinal nodes, the oldest and most basal extinct stem taxa of each afrotherian order were sampled, most of which are Eocene in age (i.e., between 55 and 34 million years old). An attempt was also made to sample most of the morphological diversity among Paleogene afrotherians, because the intraordinal relationships among such taxa remain controversial [44,49,50]. All of the extinct ingroup taxa are widely considered to be stem members of afrotherian orders with the exception of *Arsinoitherium*, which has been placed as a stem proboscidean, stem tethytherian, or stem paenungulate [33,42,43]. In unconstrained analyses, the oldest undoubted aardvark (*Myorycteropus*) consistently emerged as the sister taxon of the armadillo (*Dasypus*), in large part because both taxa retain single-rooted anterior premolars that have been lost (and thus cannot be scored) in the extant aardvark *Orycteropus*. As *Myorycteropus *otherwise exhibits a number of potentially informative character states that have either been lost or transformed in the highly derived genus *Orycteropus*, the two were constrained as sister taxa in all analyses. Outgroup taxa include the extant marsupial *Didelphis*, the Early Cretaceous stem placentals *Montanalestes *[77] and *Prokennalestes *[78,79], the xenarthran *Dasypus*, the extant euarchontans *Lemur *(order Primates) and *Tupaia *(order Scandentia), and the extant eulipotyphlans *Condylura *(family Talpidae) and *Erinaceus *(family Erinaceidae). While it is not necessarily the case that the extant outgroups adequately capture the primitive morphotype for Boreoeutheria, when compared with stem placentals these taxa are, in many ways, more generalized than the highly specialized members of other boreoeutherian clades that could have been sampled (such as Chiroptera, Perissodactyla, Pholidota, Lagomorpha, and Rodentia).

A number of extinct taxa from the Paleocene and Eocene of Laurasia (i.e., phenacodontids, phenacolophids, hyopsodontids, and apheliscids) have recently been identified as afrotherians in phylogenetic analyses with relatively limited character and/or taxon sampling [16,38,80], and these results have been interpreted as providing evidence for a Laurasian, rather than Afro-Arabian, origin of crown Afrotheria. The morphological support for such hypotheses is weak, however, and the best evolutionary explanation for the detailed morphological similarities shared by afrotherian paenungulates and laurasiatherian ferungulates remains convergent acquisition in isolation, rather than in sympatry [19]. One obvious possibility is that some or all of the aforementioned extinct laurasian taxa are in fact laurasiatherians, and this hypothesis was recently supported by Wible et al.'s [17] relatively character-rich phylogenetic analysis which found *Hyopsodus*, *Meniscotherium*, and *Phenacodus *form a sister clade of Cetartiodactyla rather than of Paenungulata (or any other afrotherian clade). These enigmatic fossil taxa are not included in the current analysis because competing hypotheses of their afrotherian versus laurasiatherian placement can only be tested through broader analyses that include more morphological characters and a large sample of living and extinct boreoeutherians (in particular perissodactyls and artiodactyls) [19].

In contrast, two enigmatic African fossil mammals with no clear link to historically Laurasian clades – early Miocene *Kelba *from east Africa and late Eocene *Widanelfarasia *from Egypt – have recently been identified as possible or probable afrotherians, respectively, based on new material [39,81]. Cote *et al*. [81] noted a few features that *Kelba *shares with aardvarks, while Seiffert *et al*. [39] have more strongly argued that *Widanelfarasia *represents a tribosphenic stem tenrecoid. Both genera exhibit unique constellations of morphological characters when compared with other living and extinct afrotherians, and as these character distributions could prove to be of use in helping to unravel afrotherian phylogeny, *Kelba *and *Widanelfarasia *were added to the matrix for secondary analyses in order to determine 1) where these taxa are placed if they are in fact afrotherians, and 2) whether their unique morphological features have an impact on afrotherian phylogeny.

## Supplementary Material

Additional file 1 **List of morphological characters**. Document contains detailed descriptions of each morphological character and character state. Click here for file

Additional file 2 **Character-taxon matrix. **Character-taxon matrix, in Nexus format, containing all nucleotide, genomic, and morphological characters used in the phylogenetic analysis. Click here for file
