# Supplementary material for: A new estimate of afrotherian phylogeny based on simultaneous analysis of genomic, morphological, and fossil evidence
Source: BMC Evol Biol. 2007 Nov 13;7:224. doi: 10.1186/1471-2148-7-224 (PMC2248600; doi:10.1186/1471-2148-7-224)
Supplement: Additional file 1 — List of morphological characters. Document contains detailed descriptions of each morphological character and character state. [file 1471-2148-7-224-S1.rtf]

ADDITIONAL FILE:  MORPHOLOGICAL CHARACTER DESCRIPTIONS

“A new estimate of afrotherian phylogeny based on simultaneous analysis of genomic, morphological, and fossil evidence”

Erik R. Seiffert, Department of Anatomical Sciences, Stony Brook University, Stony Brook, New York, 11794-8081, U.S.A.


Lower dentition/general dental characters (1-85)

1. Relative size of first and second lower incisors [MSO (=multistate ordered)], contains information similar to that of Frost et al.'s [1] character 46 and Asher's [2] character 37). Three states are recognized: first lower incisor is much smaller than second lower incisor (state 0), polymorphic expression of states 0 and 2 (state 1), first lower incisor is approximately equal in size to second lower incisor (state 2), polymorphic expression of states 2 and 4 (state 3), or first incisor is much larger than adjacent incisor (state 4). As with some other dental characters in the matrix (particularly incisor and premolar characters), it is difficult to determine positional homology across distantly related taxa (e.g., comparing the “lower first incisors” of generalized crown placentals bearing three lower incisors with those of marsupials or stem placentals that retain four lower incisors). With respect to marsupial-placental incisor homologies, unless there is clear developmental or paleontological evidence for an alternative interpretation, I have chosen to recognize the first tooth distal to the midline as being the “first lower incisor”, the following tooth as the “second lower incisor”, etc.; given this simplifying assumption, the incisor mesial to the lower canine in Didelphis and the stem placental Asioryctes (the I4) is not considered to have a homologous counterpart in crown placentals. This determination may not be correct and must be considered as a possible source of phylogenetic inaccuracy within basal nodes of resulting cladograms.

2. i1 presence. The first lower incisor is either present (state 0) or absent (state 1).

3. Accessory cusps on first lower incisor. Taxa with spatulate or lanceolate incisors that exhibit only a single occlusal edge are assigned state 0 (“unicuspid”); taxa with a single small basal accessory cusp are assigned state 1; taxa with two roughly subequal cusps on the first lower incisor are assigned state 2 (“bicuspid”); taxa with three cusps (such as most hyracoids) are assigned state 3 (“tricuspid”). The late Eocene hyracoid Antilohyrax has more than three cusps on the first lower incisor [3], and is scored as 4. Paleogene proboscideans tend to exhibit fine serrations along the distal occlusal margin; these taxa are scored as 5.

4. Shape of lower central incisor crown. Taxa with lower central incisor crowns that have a straight mesial edge, a gently curved distal surface, and a well-defined mesial apex are scored as being “lanceolate” (state 0). If the crown of the central lower incisor is buccolingually compressed and has an elongate occlusal edge -- regardless of whether that occlusal edge has serrations or accessory cusps -- that is of roughly equal height above the root at the mesial and distal aspects of the occlusal surface, the taxon is scored as being “spatulate” (state 1). Taxa with small and simple lower incisor crowns that are not notably wider than the incisor root, are not clearly “spatulate” or “lanceolate”, and that have little or no occlusal morphology, are scored as being “peg-like” (state 2). Many proboscideans with relatively massive, elongate incisor crowns are assigned state 3 (“tusk-like”). Some afrotherians have lower central incisors that are best described as caniniform (state 4). The strepsirrhine primate Lemur has lower incisors that form an elongate toothcomb (state 5), a condition not seen in any other ingroup taxa.

5. i2 presence. The second lower incisor is either present (state 0) or absent (state 1).

6. Accessory cusps on second lower incisor. Character states are the same as those for character 3. The number of accessory cusps on the second incisor is not necessarily correlated with the number of cusps on the first incisor (character 3), because certain taxa (e.g., Herodotius) exhibit different character states for characters 3 and 6.

7. Shape of second lower incisor root. The second lower incisor root is either approximately round in cross-section (state 0); suboval, being slightly compressed buccolingually (state 1); strongly compressed buccolingually and distinctly oval in cross-section (state 2); or strongly compressed mesiodistally (state 3).

8. Shape of second lower incisor. Character states are same as for character 4, with the exception of an additional state (6), “incisor crown tapers toward occlusal edge”, created for taxa such as Bunohyrax and Megalohyrax, whose incisor crowns taper to a point (with mesial and distal cusps converging on central cusp), and state 7, “premolariform”, for the condition observable in Erinaceus, which is different from all other ingroup taxa.

9. i3 presence (MSO). The third lower incisor is either present (state 0) or absent (state 2). Polymorphic expression of states 0 and 2 is scored as state 1. 

10. Accessory cusps on third lower incisor (MSO). Variation within the ingroup can be accommodated by three states: unicuspid (state 0), two cusps (bicuspid, state 1), or multiple cusps or tines (state 2).

11. Diastema between second and third lower incisor. Some Paleogene hyracoids exhibit a specialized condition in which there is a diastema that is as long as, or longer than, the mesiodistal length of the third lower incisor present between the second and third lower incisors (state 1). Most afrotherians lack such a diastema (state 0).

12. i4 presence. The fourth lower incisor is either present (state 0) or absent (state 1).

13. Diastema between ultimate lower incisor and canine (MSO). Among afrotherians there may be no diastema between the ultimate lower incisor and the canine, or a very short diastema that is shorter than the length of the lower canine (state 0), a diastema that is longer than the length of the lower canine, but less than two times the length of the lower canine (state 2), or a diastema that is at least two times the length of the lower canine (state 4). Polymorphic expression of states 0 and 2, and 2 and 4, are scored as states 1 and 3, respectively.

14. Lower canine presence (MSO). The lower canine is either present (state 0) or absent (state 2). Polymorphic expression of states 0 and 2 is scored as state 1.

15. Lower canine root number. Contains information similar to that of Domning's [4] character 144. The lower canine is either one-rooted (state 0) or two-rooted (state 2). I have followed Gheerbrant et al.'s [5] tentative suggestion that the early Eocene proboscidean Phosphatherium retains a one-rooted lower canine, and that p1 is lost.

16. Lower canine size (relative to adjacent premolar) (MSO). The lower canine is either clearly smaller than the adjacent premolar (state 0), is of approximately the same size as the adjacent premolar (state 2), or is much larger than the adjacent premolar (state 4). Polymorphic expression of states 0 and 2, and 2 and 4, are scored as states 1 and 3, respectively.

17. Diastema between lower canine and adjacent premolar (MSO). There may be no diastema separating the lower canine from the adjacent premolar (state 0), a small diastema that is at least half as long, or equal in length to, the total mesiodistal length of the lower canine (state 2), or a larger diastema that is much longer than the mesiodistal length of the lower canine (state 4). Polymorphic expression of states 0 and 2, and 2 and 4, are scored as states 1 and 3, respectively.

18. p1 presence. This and other lower premolar presence/absence characters contain information similar to that of Asher's [2] character 40. p1 is either present (state 0) or absent (state 1). For this and other premolar presence/absence characters, I have chosen to score as "present" only those premolars that are replacement teeth present during adulthood (as in most placental mammals), or if they are deciduous teeth that are retained throughout adulthood (as is often the case with the deciduous first premolar of placental mammals, and the premolars of marsupials, aside from the upper and lower “third” premolars). Extant Trichechus exhibits a pattern of horizontal tooth replacement in which primary and supernumerary molars migrate mesially along the toothrow throughout life, so the dentition of most museum specimens is made up entirely of molariform teeth. Although premolariform teeth are present in newborn manatees, Domning and Hayek [6] have argued that these teeth are homologues of the three posterior deciduous premolars of other placental mammals. Because manatees have no permanent premolars in the upper and lower dentition, and the deciduous premolars that are present are lost early in life, I score Trichechus as lacking permanent premolars in this and other similar characters (I also score Trichechus as lacking upper and lower incisors, which are present in newborn animals but shed early in life). The upper and lower premolar number of Orycteropus is open to debate -- for instance, aardvarks may well have lost their last molars (as have extant macroscelideans). However, it has been argued that the Miocene tubulidentate Myorycteropus had at least four premolars in addition to three molars [7], so I have scored premolar characters for Orycteropus with the assumption that the last tooth in its upper and lower dentition is homologous with the upper and lower third molar of other placentals.
There is now clear evidence from the Cretaceous fossil record that many stem, and possible crown, placentals retained five premolars [8-14]. Although there has been considerable debate surrounding the homology of the “fifth” premolar [15], it is now generally agreed that the tooth that has been lost is the third of five (that is, p3 if the five teeth are numbered p1-p5) [16]. Cifelli [16] suggested that this tooth be referred to as premolar “x” so that the traditional practice of numbering placental premolars p1 to p4 can be maintained, and I have followed his suggestion herein. Homologizing premolar loci prior to analysis has a number of advantages, such as the ability to score premolar features in fossil taxa when dental formula cannot be confidently determined due to breakage (e.g., if a species is represented by a maxilla with only P3 and P4 preserved, at least one can confirm that these teeth are present and not absent). This practice might lead to spurious results if the assumed homologies are incorrect, and some workers have tried to avoid this problem by using multistate characters describing “number of lower antemolars” or “number of lower premolars” in their phylogenetic analyses [2, 17], but these analyses generally also include other characters that implicitly homologize premolar teeth (by scoring features on, for instance, the “penultimate” or “ultimate” premolar, which is no different than calling these teeth “p3” and “p4”). As there is no agreement as to what the premolar homologies of marsupials and placentals might be, I have opted to score all premolar characters in the marsupial Didelphis as “missing”.

19. p1 root number (MSO). Among afrotherians the first premolar is either one-rooted (state 0), two-rooted (state 2), three-rooted (state 4), or four-rooted (state 6). Ideally this character might be best combined with character 18 in order to avoid the problem of inapplicable data, but root number in lower teeth is often very difficult to determine, particularly in fossil material. In order to maximize information content and avoid situations where taxa could not be scored for presence/absence of p1 simply because root number could not be determined, these features were not scored together as a composite character. Polymorphic expression of states 0 and 2 is scored as state 1; polymorphic expression of states 2 and 4 is scored as state 3; polymorphic expression of states 4 and 6 is scored as state 5.

20. p1 paraconid (MSO). The paraconid is the most mesial cusp on the trigonid of molars or submolariform/molariform premolars. The first lower premolar may have no, or only a very small, paraconid (state 0), a paraconid that is present but small (state 2), or a paraconid that is present, large, and distinct (state 4). Polymorphic expression of states 0 and 2 is scored as state 1; polymorphic expression of states 2 and 4 is scored as state 3.

21. p1 metaconid (MSO). The metaconid is the cusp lingual to the primary (protoconid) cusp on lower premolars. In the taxon sample considered here, the p1 metaconid is either absent or indistinct (state 0) or present but small relative to the protoconid (state 1).

22. p1 entoconid. The entoconid is a cusp that develops lingual to the hypoconid on submolariform/molariform lower premolars. The size of entoconid is scored relative to that of the hypoconid (it is assumed that the hypoconid is the first cusp to appear on the talonid of afrotherians). The entoconid may be absent (state 0) or present but smaller than hypoconid (state 1).

23. p2 presence. p2 is either present (state 0) or absent (state 2). Polymorphic expression of states 0 and 2 is scored as state 1.

24. p2 root number (MSO). The lower second premolar is either one-rooted (state 0), two-rooted (state 2), three-rooted (state 4), or four-rooted (state 6). Polymorphic expression of states 0 and 2, 2 and 4, and 4 and 6 is scored as state 1, 3, and 5 respectively.

25. p2 paraconid (MSO). Contains part of the variation that is described by Asher's [2] character 49. The paraconid on the second lower premolar is either absent or very small (state 0), present but small (state 2), or large and distinct (state 4). Polymorphic expression of states 0 and 2 is scored as state 1; polymorphic expression of states 2 and 4 is scored as state 3.

26. p2 metaconid (MSO). The metaconid is either absent or indistinct (state 0), present but small relative to the protoconid (state 2), or present and subequal in size to the protoconid (state 4). Polymorphic expression of states 0 and 2 is scored as state 1; polymorphic expression of states 2 and 4 is scored as state 3.

27. p2 hypoconid (MSO). The hypoconid is the primary cusp on the talonid of submolariform/molariform lower premolars. The size of the hypoconid is scored based on its size relative to the protoconid; it is either very small or absent (state 0), present but less than half the height of the protoconid (state 2), or present and greater than half the height of the protoconid (state 4). Polymorphic expression of states 0 and 2 is scored as state 1; polymorphic expression of states 2 and 4 is scored as state 3.

28. p2 entoconid (MSO). The entoconid is either absent (state 0), present but smaller than hypoconid (state 2), or present and subequal in size to the hypoconid (state 4). Polymorphic expression of states 0 and 2 is scored as state 1; polymorphic expression of states 2 and 4 is scored as state 3.

29. p3 paraconid (MSO). The paraconid on p3 is either absent or very small (state 0), present but small (state 2), or large and distinct (state 4). Polymorphic expression of states 0 and 2 is scored as state 1; polymorphic expression of states 2 and 4 is scored as state 3.

30. p3 metaconid (MSO). The metaconid may be absent or indistinct (state 0), present but small relative to the protoconid (state 2), or present and subequal in size to the protoconid (state 4). Polymorphic expression of states 0 and 2 is scored as state 1; polymorphic expression of states 2 and 4 is scored as state 3.

31. p3 hypoconid (MSO). The hypoconid is either indistinct or absent (state 0), present but less than half the height of the protoconid (state 1), or present and greater than half the height of the protoconid (state 2).

32. p3 entoconid (MSO). The entoconid may be absent (state 0), present but smaller than hypoconid (state 2), or present and subequal in size to the hypoconid (state 4). Polymorphic expression of states 0 and 2 is scored as state 1; polymorphic expression of states 2 and 4 is scored as state 3.

33. p3 stylids. Some afrotherians have a sharp, distally protruding stylid that is placed on the distal wall of the protoconid (a “protostylid”, state 0), or on the distal side of the metaconid (a “metastylid”, state 1). These stylids are generally absent (state 0).

34. p3 root number (MSO). The lower third premolar is either one-rooted (state 0), two-rooted (state 1), three-rooted (state 2), or four-rooted (state 3).

35. p4 paraconid (MSO). The paraconid on the fourth lower premolar is either absent or very small (state 0), present but small (state 1), or large and distinct (state 3). Polymorphic expression of states 1 and 3 is scored as state 2.

36. p4 metaconid (MSO). The metaconid may be absent or indistinct (state 0), present but small relative to the protoconid (state 1), or present and subequal in size to the protoconid (state 2).

37. p4 protolophid (MSO). On molarized lower premolars, the two primary trigonid cusps (the protoconid and metaconid) are either isolated and not connected by a transverse crest (state 0), connected by an incipient protolophid that is depressed and not horizontally continuous (state 2), or connected by a tall protolophid that is horizontally continuous and incorporates the two cusps into an extensive shearing edge (state 4). Polymorphic expression of states 0 and 2 is scored as state 1; polymorphic expression of states 2 and 4 is scored as state 3.

38. p4 premetacristid (MSO). Some afrotherians have a crest that runs along the mesial face of the metaconid, in some cases helping to enclose the trigonid basin lingually. Taxa with such a premetacristid on p4 are assigned state 2; if a premetacristid is absent the taxon is assigned state 0. Polymorphic expression of states 0 and 2 is scored as state 1.

39. p4 entoconid (MSO). The entoconid may be absent (state 0), present but smaller than hypoconid (state 2), or present and subequal in size to the hypoconid (state 4). Polymorphic expression of states 0 and 2 is scored as state 1; polymorphic expression of states 2 and 4 is scored as state 3.

40. p4 hypoconid (MSO). The hypoconid is either indistinct or absent (state 0), present but less than half the height of the protoconid (state 1), or present and greater than half the height of the protoconid (state 2).

41. p4 hypolophid (MSO). On molarized lower premolars, the two primary talonid cusps (the entoconid and hypoconid) are either isolated and not connected by a transverse crest (state 0), connected by an incipient hypolophid that is depressed and not horizontally continuous (state 2), or connected by a tall hypolophid that is horizontally continuous and incorporates the two cusps into an extensive shearing edge (state 4). Polymorphic expression of states 0 and 2 is scored as state 1; polymorphic expression of states 2 and 4 is scored as state 3.

42. Position of metaconid relative to protoconid on p4 (MSO). Among afrotherians the p4 metaconid is either situated directly transverse to the protoconid (state 0), or it is situated distal to the protoconid (state 2). Polymorphic expression of states 0 and 2 is scored as state 1.

43. Position of hypoconid on p4 (MSO). The hypoconid can take one of three positions on the p4 talonid: either lingually placed (state 0), centrally placed (state 1), or buccally placed (state 2) relative to the midline of the trigonid.

44. Cristid obliqua terminus on p4. The cristid obliqua is a crest that connects the hypoconid to the posterior wall of the trigonid on lower molars and premolars. In its mesial termination on the distal wall of the trigonid, it can either meet the apex of the metaconid (state 0), terminate approximately midway between the protoconid and metaconid (state 2), or meet the apex of the protoconid (state 4). In some taxa the cristid obliqua is indistinct or absent (state 5). Polymorphic expression of states 0 and 2 is scored as state 1; polymorphic expression of states 2 and 4 is scored as state 3.

45. Relative width of talonid basin on p4 (MSO). The talonid basin on the lower fourth premolar is either distinctly narrower than the p4 trigonid (state 0), subequal in width to the p4 trigonid (state 2), or distinctly wider than the p4 trigonid (state 4). Polymorphic expression of states 0 and 2 is scored as state 1; polymorphic expression of states 2 and 4 is scored as state 3.

46. Metaconid shape on p4. In some taxa (e.g., some macroscelideans), the p4 metaconid is tall and maintains a roughly tubular or columnar shape from its base to its occlusal edge (state 0). In most other taxa, the mesial and distal walls of the p4 metaconid flare gradually towards the base of the cusp, and are more pyramidal in shape (state 1).

47. p4 root number (MSO). Either one-rooted (state 0), two-rooted (state 1), three-rooted (state 2), or four-rooted (state 3).

48. Lower premolar “x”. Contains information similar to that of Domning's [4] character 146. Unlike all other generalized crown placentals, the most primitive known fossil sirenians have five premolars instead of four. Unfortunately the homologies of early sirenian premolars have not been convincingly worked out; in this study I treat the supernumerary premolar as the third premolar of five in the tooth row, as appears to have been the case in primitive stem placentals [16]. The ultimate premolar in the dentition of fossil sirenians is scored as if it were homologous with the p4 of other afrotherians, the penultimate tooth as homologous with p3, etc. Taxa that retain, or have secondarily evolved, px are assigned state 1; taxa that have lost px are assigned as state 0.

49. Length of p4 relative to m1 (MSO). Character states are expressed as ranges of ratios (maximum mesiodistal length of p4 relative to the maximum mesiodistal length of m1): taxa with a lower fourth premolar that is longer than the first molar are assigned state 0, taxa with p4s that are 90-100% the length of m1 are assigned state 2; taxa with p4s that are 80-89% the length of m1 are assigned state 4, and taxa with p4s that are 80% the length of m1 (or shorter) are assigned state 6. Polymorphic expressions of these states are scored as states 1, 3, and 5, respectively.

50. m1 area (MSO). Areas of the first lower molar are used as a rough proxy for body mass -- character states are divided between taxa that have m1 areas between 0 and 50 mm2 (state 0), 51-100 mm2 (state 2), 101-150 mm2 (state 4), 151-200 mm2 (state 6), 201-250 mm2 (state 8), 251-300 mm2 (state A), and 300 mm2 and above (state G). Polymorphic expressions of these states are scored as states 1, 3, 5, 7, 9, and C, respectively.

51. m1 area/m2 area (MSO). Most afrotherians have first and second lower molars that are either roughly equal in size, or an m2 that is slightly larger than m1 - taxa that fall within a range between equality and an m1/m2 ratio greater than .65 are scored as state 2; taxa with an m1 that is larger than m2 are scored as state 0; taxa in which m1 area is less than 65% the size of m2 (as in some early proboscideans) are scored as state 4. Polymorphic expressions of states 0 and 2 are scored as state 1; polymorphic expressions of states 2 and 4 are scored as state 3.

52. m1 roots (MSO). The first lower molar is either one-rooted (state 0), two-rooted (state 1), or four-rooted (state 2).

53. Lower molar mesoconids/”protoconulids”. A mesoconid is a small accessory cuspule that can develop on the cristid obliqua of lower molars. Most afrotherians lack this feature (state 0); taxa that exhibit mesoconids on the lower molars are assigned state 1. Some elephantiform proboscideans (i.e., Phiomia, Palaeomastodon, and later forms more closely related to extant elephants) exhibit large cusps in the general vicinity of the cristid obliqua (of which there is no clear remnant), but there is no evidence from the fossil record that these enlarged cusps originated along the cristid obliqua as a mesoconid. Here primitive elephantiforms are scored as having enlarged “protoconulids” (state 2), and this multi-state character is treated as unordered.

54. Development of postmetacristids on lower molars (MSO). Postmetacristids are crests on the distal face of lower molar or premolar metaconids, and can take the form of simple trenchant crests (state 2), or well-developed metastylids (state 4). Most afrotherians lack any development of postmetacristids on the lower molars (state 0). Polymorphic expression of states 0 and 2 is scored as state 1; polymorphic expression of states 2 and 4 is scored as state 3.

55. m1-2 trigonid height relative to talonid (MSO). Primitive fossil placentals exhibit a considerable height discrepancy between an elevated trigonid and a low, poorly developed talonid on the lower molars (state 0). In many crown placentals the trigonid is taller than the talonid but this discrepancy is not quite so marked as in stem placentals (state 2). A number of afrotherians (e.g., most paenungulates) have trigonids that are roughly equal in height to the molar talonids (state 4). Polymorphic expression of states 0 and 2 is scored as state 1; polymorphic expression of states 2 and 4 is scored as state 3.

56. Presence/absence of spurs in trigonid basins (MSO). A number of Paleogene hyracoids have small spurs that descend from the faces of the molar metaconid and protoconid into the trigonid basin. These are not scored as paracristids or premetacristids because they can occur alongside such crests. Taxa that have such spurs are scored as state 2, taxa without trigonid spurs are scored as 0. Polymorphic expressions of states 0 and 2 are scored as state 1.

57. Molar paraconid presence/paracristid length on m1-2 (MSO). Judging from the morphology observable in stem placentals, the primitive condition within Placentalia is likely the presence of a well-developed paraconid that is connected to the protoconid by a trenchant paracristid (state 0). Afrotherians exhibit at least two intermediate states between state 0 and state 6 (which is assigned to taxa with no paraconid and no clear development of a paracristid): state 2 -- long paracristid, no cuspidate paraconid, and state 4 - short paracristid, no cuspidate paraconid. Polymorphic expression of states 0 and 2 is scored as state 1; polymorphic expression of states 2 and 4 is scored as state 3; polymorphic expression of states 4 and 6 is scored as state 5.

58. Morphology of lower molar paracristids. Some “insectivores”, such as tenrecs and golden moles, have paracristids that do not course gradually across the front of the trigonid, but are discontinuous and somewhat notched, descending approximately mesially from the face of the protoconid, and then meeting a crest that courses lingually toward the paraconid (state 1); in other taxa the paracristid is not notched and courses gradually across the mesial aspect of the trigonid (state 0).

59. Height of molar metaconids relative to protoconids (MSO). Among afrotherians, taxa either have molar metaconids that are lower than protoconids (state 0), metaconids and protoconids that are of roughly equal height (state 2), or metaconids that are elevated relative to protoconids (state 4). Polymorphic expressions of states 0 and 2 are scored as state 1; polymorphic expressions of states 2 and 4 are scored as state 3.

60. m1-2 entoconid (MSO). Molar entoconids are a consistent component of the talonid in tribosphenic mammals, and are almost certainly plesiomorphic within Placentalia given their presence in stem placentals such as Eomaia [10] and Montanalestes [18]. They are present in most afrotherians (state 0). In some incipiently zalambdodont taxa (that is, taxa whose molars are intermediate between typical tribosphenic forms and zalambdodont forms that have lost the metacone and talonid altogether), the entoconid is much reduced and crestiform (state 1). Zalambdodonts have no cuspidate entoconid (state 2).

61. m1-2 hypoconulid. Among afrotherians, lower molars either exhibit cuspidate hypoconulids (state 0), hypoconulids that are reduced and crestiform (state 1), no hypoconulids but a “peaked” distal cingulid (state 2 -- distal cingulid rises toward the occlusal surface of the molar, as in most Paleogene hyracoids; in some taxa the cingulid is occasionally connected to the hypoconid), or there is no trace of a hypoconulid on the lower molars (state 3).

62. m1-2 hypoconid. Almost all afrotherians have lower molar hypoconids; these are either tall and well-developed (state 0 - also scored for taxa that have hypoconids and entoconids incorporated into hypolophids, despite the fact that the cusps are not strictly “cuspidate”), or reduced, crestiform, or absent (state 1).

63. Relative length of m1-2 talonid (MSO). Contains information similar to that of Asher's [2] character 44. Among afrotherians the talonid varies considerably in relative length, being either totally absent (state 0) or present but shorter than the trigonid (state 2) in some zalambdodont forms, to being present and subequal in length to the trigonid (state 4), to being longer than the trigonid (state 6). Polymorphic expression of states 0 and 2 is scored as state 1; polymorphic expression of states 2 and 4 is scored as state 3; polymorphic expression of states 4 and 6 is scored as state 5.

64. m1 postentoconulid (MSO). The postentoconulid is an accessory cusp that develops lingual to the hypoconulid on the first lower molar of some paenungulates, and is absent in most taxa (state 0). When present, the postentoconulid can be isolated and not connected to the hypoconulid (state 2), or connected to the hypoconulid via a third lophid distal to the hypolophid (state 4). Polymorphic expression of states 0 and 2 is scored as state 1; polymorphic expression of states 2 and 4 is scored as state 3.

65. Lower molar buccal cingulids (MSO). Among afrotherians, buccal cingulids are either totally absent (state 0), present but poorly-developed and not continuous across the buccal face of the lower molars (state 2), or well-developed and continuous (state 4). Polymorphic expressions of states 0 and 2 are scored as state 1; polymorphic expressions of states 2 and 4 are scored as state 3.

66. Lower molar precingulids (MSO). Variation in the expression of precingulids can be encompassed by two character states -- present and distinct (state 2) or absent or indistinct (state 0). Polymorphic expressions of states 0 and 2 are scored as state 1.

67. Orientation of buccal m1-2 trigonid walls (MSO). The buccal face of the protoconid may be basally inflated, which leads the cusp to be relatively internally placed (state 2), or there may be no basal inflation of this cusp and consequently a vertically-oriented buccal face of the protoconid (state 0). Polymorphic expressions of states 0 and 2 are scored as state 1.

68. Orientation of lingual m1-2 trigonid walls (MSO). Scoring of this character is the same as in character 67, but for the metaconid cusps. Characters 67 and 68 appear to be independent because some taxa exhibit basal inflation of buccal cusps but no basal inflation of lingual cusps.

69. Bilateral posttrigonid constriction on lower molars (MSO). Tubulidentates are unique among afrotherians in having a bilateral constriction distal to the presumed distal wall of the trigonid (state 2). Almost all other afrotherians have clear hypoflexids buccally for occlusion of the paracone, but none have a constriction lingually (state 0). Polymorphic expressions of states 0 and 2 are scored as state 1.

70. Depth of hypoflexids on m2. The hypoflexid is the variably developed fossa buccal to the cristid obliqua that serves as an occlusal basin for the paracone of the occluding upper molar. In some afrotherians the hypoflexid is relatively shallow (state 0) - in these taxa, most of the space distal to the postcristid and mesial to the hypoconid is composed of talonid basin defined labially by the cristid obliqua. In zalambdodonts and incipiently zalambdodont taxa, the hypoflexid is very deep, and most of space distal to postcristid and mesial to hypoconid is labially open for occlusion with the paracone (state 1).

71. Cristid obliqua morphology on lower molars. Among afrotherians the cristid obliqua on the lower molars either ascends the posterior face of the trigonid and encloses the entire buccal margin of the talonid (state 0), or it descends abruptly from the hypoconid and terminates in the talonid basin of the lower molar (state 1). In some taxa the cristid obliqua is poorly defined or absent (state 2).

72. Cristid obliqua orientation on m1-2 (MSO). The cristid obliqua either terminates behind the apex of the metaconid (state 0), midway between the metaconid and protoconid (state 2), or behind the apex of the protoconid (state 4). Polymorphic expressions of states 0 and 2 are scored as state 1; polymorphic expressions of states 2 and 4 are scored as state 3.

73. Shape of buccal wall of hypoconid (MSO). The hypocristid and the cristid obliqua either meet at a sharp angle, forming a “v”-shaped buccal wall of the hypoconid (state 0), or this junction is gently rounded (state 2). Polymorphic expressions of states 0 and 2 are scored as state 1.

74. Morphology of lower molar protocristids (MSO). Among afrotherians molar protocristids are either discontinuous and deeply notched centrally (state 0), are complete but depressed, with the protoconid and metaconid standing well above the central depression of the protocristid (state 2), or form a tall, horizontally continuous protolophid (state 4). Polymorphic expression of states 0 and 2 is scored as state 1; polymorphic expression of states 2 and 4 is scored as state 3.

75. Morphology of crest connecting hypoconid and entoconid. The distal crests of the talonid exhibit some interesting peculiarities in afrotherians -- there may be no crests connecting the entoconid and hypoconid (state 0 - this state is assigned to forms with typical tribosphenic teeth that have a hypoconulid situated between hypoconid and entoconid, and to forms with no hypoconulid); the crest connecting the hypoconid and entoconid can be incipient, being poorly-developed and depressed (state 1); the crest can be tall and well-developed, forming a tall hypolophid that connects the two cusps (state 3). An alternative state (state 4) is defined for some primitive macroscelideans (e.g., Metoldobotes) that have a crest that courses lingually from the hypoconid but only meets the base of the entoconid. Polymorphic expression of states 1 and 3 is scored as state 2.

76. Morphology of crest connecting hypoconid and hypoconulid (or "peaked" postcingulid) on m1-2 (MSO). Taxa with typical tribosphenic molars either have short trenchant crests that connect the hypoconid and hypoconulid (state 0) or crests that are present but weak (state 2). A number of Paleogene afrotherians have a trenchant crest that courses from the hypoconid to a peaked postcingulid, and this state is scored as state 0. Other taxa have no crest connecting the hypoconid and hypoconulid (state 4). Polymorphic expression of states 0 and 2 is scored as state 1; polymorphic expression of states 2 and 4 is scored as state 3.

77. Lower molar entocristids. Entocristids are crests that develop on the mesial face of the entoconid and enclose the lingual aspect of the talonid; they are scored as being either present and trenchant (state 2) or absent/indistinct (state 0). Polymorphic expressions of states 0 and 2 are scored as state 1.

78. Lower molar premetacristids (see character 38 for definition of “premetacristid”). Premetacristids are either present and trenchant on lower molars (state 2), or absent/indistinct (state 0). Polymorphic expressions of states 0 and 2 are scored as state 1.

79. Protocristid orientation on lower molars (MSO). The protocristid is either slightly oblique with respect to the long axis of the tooth row, with the metaconid position mesial to the protoconid (state 0); the protocristid is oriented transversely, with the metaconid and entoconid positioned opposite each other and the crest trending approximately perpendicular to the long axis of the toothrow (state 2); the protocristid is slightly oblique, but, in contrast to state 0, the metaconid is situated distal to the protoconid (state 4); or the protocristid is strongly oblique, with the metaconid shifted far distal to the protoconid (state 6). Polymorphic expressions of these states are scored as states 1, 3, and 5, respectively.

80. Shape of m3 hypoconulid lobe (MSO). There is considerable variation in the morphology of the m3 talonid among afrotherians; taxa considered here either lack a hypoconulid (state 0), have a small hypoconulid that projects from the distal margin of the talonid (state 2), have a large lobate hypoconulid structure that is unicuspid (state 4), or have a large lobate hypoconulid that is situated next to a postentoconulid (state 6). Polymorphic expressions of states 0 and 2 are scored as state 1; polymorphic expressions of states 2 and 4 are scored as state 3; polymorphic expression of states 4 and 6 is scored as state 5.

81. Size/presence of m3 (MSO). Contains information similar to that of Asher's [2] character 46. There is considerable variation in the size of m3 relative to m2, with some taxa having an m3 that is considerably larger than m2 (state 0), other taxa having an m3 that is approximately equal in size to m2 (state 2), and some taxa having an m3 that is smaller than m2 (state 4). Some taxa (e.g., crown macroscelideans) have lost m3 (state 6). Polymorphic expressions of states 0 and 2 are scored as state 1; polymorphic expressions of states 2 and 4 are scored as state 3; polymorphic expression of states 4 and 6 is scored as state 5.

82. m3 root number (MSO). The last lower molar is either one-rooted (state 0), two-rooted (state 2), four-rooted (state 4), or has five (or more) roots (state 6). Polymorphic expressions of states 0 and 2 are scored as state 1; polymorphic expression of states 2 and 4 is scored as state 3; polymorphic expression of states 4 and 6 is scored as state 5.

83. Lower fourth molar. Living and extinct placentals are differentiated from marsupials such as Didelphis by having only three unreplaced molariform teeth (state 1); primitively, crown marsupials have four unreplaced molariform teeth (state 0).

84. Presence/absence of tubulidentine. Tubulidentates are unique among afrotherians in having tubulidentine (state 1); all other ingroup taxa lack this peculiar apomorphy (state 0).

85. Presence/absence, and morphology of, enamel on molars (MSO). Contains information similar to that of Domning's [4] character 156. With the exception of aardvarks, which have no molar enamel (state 4), all afrotherians have molar enamel, although it differs in thickness and morphology. Among afrotherians, molar enamel can be extremely thin and smooth (state 3), relatively thick and smooth (state 2), relatively thick and crenulated (state 1), or relatively thick with numerous accessory cuspules (state 0).

Upper dentition (86-164)

86. Interincisal diastema (MSO). A number of afrotherians have a diastema separating the central upper incisors. This diastema can be relatively narrow (about the mediolateral width of a single upper central incisor, state 1), or relatively wide (much wider than the width of a single upper central incisor, state 2). Some taxa lack a diastema (incisors contact in the midline) or have a very small diastema that is more narrow than the width of a single central upper incisor (state 0).

87. I1 presence. The first upper incisor is either present (state 0) or absent (state 1).

88. Relative size of upper central incisors (MSO). Contains information similar to that of Asher's [2] character 36. Upper central incisors are either much larger than the adjacent incisor (state 0), of approximately the same size as the adjacent incisor (state 1), or much smaller than the adjacent incisor (state 2). The relative size of the upper central incisors is independent of the size of the lower central incisors, as taxa with relatively large and relatively small lower central incisors can have either relatively large or relatively small upper central incisors.

89. Diastema between central and adjacent incisors (MSO). Some afrotherians have a closely packed upper incisor dentition in which there is either no diastema between the central and adjacent incisors, or only a very small diastema that is shorter than the mesiodistal length of the adjacent incisor (state 0); other taxa have a relatively wide diastema that is either subequal in length to the mesiodistal length of the adjacent incisor (state 1) or much longer than the mesiodistal length of the adjacent incisor (state 2).

90. I2 presence. The second upper incisor is either present (state 0) or absent (state 2).

91. I2 roots (MSO). The second upper incisor is either single-rooted (state 0) or double-rooted (state 2). Polymorphic expressions of states 0 and 2 are scored as state 1.

92. I3 presence (MSO). The third upper incisor is either present (state 0) or absent (state 2). Polymorphic expressions of states 0 and 2 are scored as state 1.

93. I3 roots (MSO). The third upper incisor is either single-rooted (state 0) or double-rooted (state 2). Polymorphic expressions of states 0 and 2 are scored as state 1.

94. I4 presence. The fourth upper incisor is either present (state 0) or absent (state 1).

95. I5 presence. The fifth upper incisor is either present (state 0) or absent (state 1).

96. Diastema between upper canine and adjacent incisor. There is either no, or only a very short, diastema between the upper canine and adjacent incisor that is shorter than the mesiodistal length of the last upper incisor (state 1), or a more extensive diastema that is longer than the mesiodistal length of the last upper incisor (state 0).

97. Upper canine presence/root number (MSO). The upper canine is either absent (state 0), present and one-rooted (state 1), present and two-rooted (state 2), or present and three-rooted (state 3).

98. Size of upper canine relative to adjacent premolar (MSO). The upper canine is either markedly smaller than the adjacent premolar (state 0), subequal in size to the adjacent incisor (state 2), or markedly larger than the adjacent premolar (state 4). Polymorphic expression of states 0 and 2 is scored as state 1; polymorphic expression of states 2 and 4 is scored as state 3.

99. Upper canine metacone presence/size. A metacone is a buccal cusp that is primitively present on tribosphenic upper molars (distal to the paracone); in many afrotherians metacones also develop on the upper premolars. Most afrotherians lack a metacone on the upper canine (state 0). If a metacone is present, it is poorly developed and closely appressed to the paracone (state 1).

100. Upper canine parastyle. A parastyle is a small accessory cusp or crest that either descends from the face of the paracone or projects from the mesial cingulum of an upper tooth. The upper canine either lacks a parastyle (state 0), or has a small parastyle (state 1).

101. Supernumerary (fifth) premolar. Some living and extinct placentals have five premolars (state 0); most have only four (state 1)

102. P1 presence. The first upper premolar is either present (state 0) or absent (state 2). Polymorphic expression of states 0 and 2 is scored as state 1.

103. P1 root number (MSO). The number of roots present in the first upper premolar varies from one (state 0), to two (state 1), to three (state 2), to four or more (state 3).

104. P1 protocone (MSO). A protocone is a cusp that is primitively present, lingual to the paracone and metacone, on the tribosphenic upper molar. In many afrotherians protocones have developed on the upper premolars. Most afrotherians lack a protocone on the first upper premolar (state 0). If a protocone is present, it is either small (state 2) or large and well-developed (state 4). Polymorphic expressions of states 0 and 2, and of 2 and 4, are scored as states 1 and 3, respectively.

105. P1 metacone (MSO). The first upper premolar generally lacks a metacone (state 0); if present, the metacone is either poorly developed and closely appressed to the paracone (state 2), or well-developed and clearly differentiated from the paracone as an independent cusp (state 4). Polymorphic expression of states 0 and 2 is scored as state 1; polymorphic expression of states 2 and 4 is scored as state 3.

106. P1 hypocone. The hypocone is a cusp that is often present on the upper molars of larger mammals with more omnivorous or herbivorous diets; in some afrotherians hypocones have developed on the upper premolars. The first upper premolar generally lacks a hypocone (state 0) or has a hypocone that is much smaller than the protocone (state 2). Polymorphic expressions of states 0 and 2 are scored as state 1.

107. P1 parastyle. The first upper premolar either lacks a parastyle (state 0), or has a small parastyle (state 1).

108. P2 presence. The second upper premolar is either present (state 0) or absent (state 1).

109. P2 root number (MSO). The number of roots present in the second upper premolar varies from one (state 0), to two (state 2), to three (state 4), to four or more (state 6). Polymorphic expression of states 0 and 2 is scored as state 1; polymorphic expression of states 2 and 4 is scored as state 3; polymorphic expression of states 4 and 6 is scored as state 5.

110. P2 protocone (MSO). The second upper premolar either lacks a protocone (state 0), or, if the cusp is present, it is either small (state 1) or large and well-developed (state 2).

111. P2 metacone (MSO). The second upper premolar either lacks a metacone (state 0), or, if present, the cusp is either poorly developed and closely appressed to the paracone (state 1), or well-developed and clearly differentiated from the paracone as an independent cusp (state 2).

112. P2 hypocone (MSO). The second upper premolar generally lacks a hypocone (state 0); if present, the P1 hypocone is either much smaller than the protocone (state 1), or well-developed and subequal in size to the protocone (state 2).

113. P2 parastyle. The second upper premolar either lacks a parastyle (state 0), or has a small parastyle (state 1).

114. P2 mesostyle. A mesostyle is a fold of enamel projecting from the buccal wall of upper molars or premolars, generally placed between the paracone and metacone at the middle of the centrocrista. Among afrotherians, the second upper premolar either lacks a mesostyle (state 0), or, if the mesostyle is present, it is small (state 1).

115. P3 protocone (MSO). Contains part of the variation that is described by Asher's [2] character 48. The third upper premolar either lacks a protocone (state 0), or, if the cusp is present, it is either small (state 2) or large and well-developed (state 4). Polymorphic expression of states 0 and 2 is scored as state 1; polymorphic expression of states 2 and 4 is scored as state 3.

116. P3 metacone (MSO). The third upper premolar either lacks a metacone (state 0), or, if present, the cusp is either poorly developed and closely appressed to the paracone (state 2), or well-developed and clearly differentiated from the paracone as an independent cusp (state 4). Polymorphic expression of states 0 and 2 is scored as state 1; polymorphic expression of states 2 and 4 is scored as state 3.

117. P3 hypocone (MSO). The third upper premolar either lacks a hypocone (state 0), or if the cusp is present, it is either much smaller than the protocone (state 2), or well-developed and subequal in size to the protocone (state 4). Polymorphic expressions of states 0 and 2 are scored as state 1; polymorphic expressions of states 2 and 4 are scored as state 3.

118. P3 paraconule (MSO). The paraconule is a small cusp on upper molars, and less commonly on upper premolars, that is generally situated buccal and mesial to the protocone, and lingual to the paracone. This cusp is either absent or indistinct (state 0), present and small (state 1), or present and large (state 2).

119. P3 metaconule (MSO). The metacone is a small cusp, usually placed directly distal to the paraconule. This cusp is either absent or indistinct (state 0), present and small (state 1), or present and large (state 2).

120. P3 parastyle (MSO). The third upper premolar either lacks a parastyle (state 0), or, if a parastyle is present, it is either small (state 1), or large and distinct (state 2).

121. P3 mesostyle. The third upper premolar either lacks a mesostyle (state 0), or, if the mesostyle is present, it is large and distinct (state 1).

122. P3 ectostyle (MSO). The ectostyle is a small cusp, present on the premolars of most tenrecs, that is generally positioned buccal and distal to the paracone, opposite the postparacrista. As it co-occurs with the stylocone in some taxa, it cannot be homologous with that cusp. It is either absent (state 0) or present (state 2); polymorphic expression of these states is scored as state 1.

123. P3 stylocone. A stylocone is a small cusp that develops on the stylar shelf of molars and premolars. In some afrotherians a stylocone is present on the stylar region of the upper third premolar (state 1); in most other ingroup taxa it is absent (state 0).

124. P3 root number (MSO). The number of roots present in the third upper premolar varies from one (state 0), to two (state 1), to three (state 2), to four or more (state 3). Taxa that have fused distal and labial roots are assigned state 1.

125. Placement of P3 distal root (MSO). In the Miocene tenrec Protenrec, the distal and buccal root of the P3 is placed mesial to the lingual root of P4 (state 1), rather than being placed more buccal and aligned with P4, as in other tenrecs and non-tenrec afrotherians (state 0).

126. P4 root number (MSO). The number of roots present in the fourth upper premolar varies from one (state 0), to two (state 1), to three (state 2), to four or more (state 3).

127. Exposure of mesial root of P4. In some afrotherians (e.g., Protenrec) the mesial face of the mesiolabial root of P4 is widely exposed (state 1); in most other afrotherians the mesiolabial root is encompassed by alveolar bone (state 0).

128. P4 protocone (MSO). The upper fourth premolar either has no protocone (state 3), is present but very small (state 2), is present but less than half the size of the paracone (state 1), or is present and greater than half the size of the paracone (state 0).

129. P4 metacone (MSO). The fourth upper premolar either lacks a metacone (state 0), or, if present, the cusp is either poorly developed and closely appressed to the paracone (state 2), or well-developed and clearly differentiated from the paracone as an independent cusp (state 4). Polymorphic expression of states 0 and 2 is scored as state 1; polymorphic expression of states 2 and 4 is scored as state 3.

130. P4 hypocone (MSO). The fourth upper premolar either lacks a hypocone (state 0), or, if the cusp is present, it is either much smaller than the protocone (state 2), or well-developed and subequal in size to the protocone (state 4). Polymorphic expressions of states 0 and 2 are scored as state 1; polymorphic expressions of states 2 and 4 are scored as state 3.

131. P4 parastyle (MSO). The fourth upper premolar either lacks a parastyle (state 0), or, if a parastyle is present, it is either small (state 2), or large and distinct (state 4). Polymorphic expression of states 0 and 2 is scored as state 1; polymorphic expression of states 2 and 4 is scored as state 3.

132. P4 paraconule (MSO). The P4 paraconule is either absent (state 0), present and small (state 1), or present and large (state 2).

133. P4 metaconule (MSO). The P4 paraconule is either absent (state 0), present and small (state 1), or present and large (state 2).

134. P4 ectostyle. The P4 ectostyle is either absent (state 0) or present (state 1).

135. P4 ectocrista (MSO). The ectocrista is a distally oriented crest that descends from the buccal face of the P4 paracone, enclosing a distobuccal “ectofossa”. The crest is either absent (state 0), present and connects with the ectostyle (state 1), or is present and does not meet the ectostyle (state 2).

136. P4 mesostyle (MSO). The fourth upper premolar either lacks a mesostyle (state 0), or, if the mesostyle is present, it is either small (state 2), or large and distinct (state 4). Polymorphic expression of states 0 and 2 is scored as state 1; polymorphic expression of states 2 and 4 is scored as state 3.

137. P4 stylocone (MSO). In some afrotherians a stylocone is present on the stylar region of the upper fourth premolar (state 1); in most taxa it is absent (state 0).

138. Height of P4 paracone. Some afrotherians (e.g., some tenrecids) have an enlarged paracone on the fourth upper premolar that forms the apex of a tall, distally oriented shearing blade; in these taxa the paracone is much taller than that on the M1 (state 0). In most other afrotherians the P4 paracone is shorter, or equal in height to, the M1 paracone (state 1).

139. Position of P4 protocone with respect to the paracone (MSO). The protocone on P4 is generally positioned directly lingual to the paracone (state 2); some taxa have a protocone that is shifted mesially with respect to the paracone (state 0), while others have a protocone that is shifted distally with respect to the paracone (state 4). Polymorphic expression of states 0 and 2 is scored as state 1; polymorphic expression of states 2 and 4 is scored as state 3.

140. Connection between hypocone and buccal cusps on P4. Among the afrotherians that have hypocones on the upper fourth premolar, the primary connection between the hypocone and the buccal cusps is either via a prehypocrista trending mesiobuccally (state 2) or a posthypocrista trending distobuccally. Among the taxa with a trenchant posthypocrista, the connection with the metacone is either via a tall crest that encloses the distal aspect of the tooth (state 0), or via a low crest (state 1).

141. Buccally oriented spurs on premolar protocones. The Eocene-Oligocene hyracoids Saghatherium and Selenohyrax have spurs that emerge from the buccal aspect of premolar protocones [19] (state 1). In all other afrotherians these spurs are absent (state 0).

142. Lingual spurs on M1-2 buccal cusps. Some Paleogene hyracoids have “spurs” of enamel that project lingually from the paracone, metacone, or the intervening area delimited by the ectoloph (state 1). Most afrotherians lack these spurs (state 0).

143. Mesial cingulum on M1-2 (MSO). Among afrotherians and outgroup taxa, the mesial cingulum on the upper first and second molars is either well-developed and continuous along the entire mesial face of the tooth (state 4), well-developed along the mesial face of the protocone, but absent labially along the mesial face of the paracone (state 2), or it is absent (state 0). Polymorphic expression of states 0 and 2 is scored as state 1; polymorphic expression of states 2 and 4 is scored as state 3.

144. Lingual cingulum around base of protocone on M1-2 (MSO). Variation in the development of the lingual cingulum on the upper first and second molar can be described by four states: either there is no cingulum around the protocone and no development of a postcingulum (i.e., a cingulum situated on the distal face of the protocone, or in the intervening space between the protocone and the hypocone) (state 0); there is no cingulum around the lingual face of the protocone, but a postcingulum is present (state 2); there is a poorly-developed cingulum on the lingual face of the protocone, and a postcingulum is present (state 4); or there is a well-developed, continuous lingual cingulum that is connected mesially to the mesial cingulum and distally to the postcingulum (state 6). Polymorphic expression of states 0 and 2 is scored as state 1; polymorphic expression of states 2 and 4 is scored as state 3; polymorphic expression of states 4 and 6 is scored as state 5.

145. Development of postprotocristae on M1-2 (MSO). The postprotocrista is a crest that courses distolabially, distally, or distolingually from the apex of protocone. Among ingroup taxa the crest is either absent (state 0), present but very weakly developed (state 2), or present and trenchant (state 4). Polymorphic expressions of states 0 and 2 are scored as state 1; polymorphic expressions of states 2 and 4 are scored as state 3.

146. Development of posthypocristae on M1 (MSO). The posthypocrista is a crest that courses distally from the apex of the hypocone. In this study it is scored as being either present and trenchant (state 2), or absent or indistinct (state 0). Polymorphic expressions of states 0 and 2 are scored as state 1.

147. Orientation of prehypocristae on M1-2. The prehypocrista is a crest that courses mesially or labially from the apex of the hypocone. It is either oriented mesially, terminating mesial to the apex of the metacone (state 0); oriented more labially, terminating along the mesial wall of the metacone (state 1); oriented labially, meeting the apex of the metacone to form a weak metaloph that is interrupted by a median furrow (state 2); or it is oriented labially and meets the metacone to form a complete metaloph (state 3).

148. Orientation of preprotocristae on M1-2 (MSO). The preprotocrista is a crest that courses mesially or labially from the apex of the protocone. Among afrotherians, the preprotocrista is either oriented mesially and is labially continuous with the parastyle, preparacrista, or the mesial cingulum (state 0); oriented mesially, terminating mesial to the mesial face of the paracone but is not continuous with the parastyle, preparacrista, or mesial cingulum (state 2); oriented more labially, meeting the mesial face of the paracone (state 4); oriented labially, forming a weak protoloph that is interrupted by a median furrow (state 6); or oriented labially, forming a strong uninterrupted protoloph (state 8). Polymorphic expressions of states 0 and 2 are scored as state 1; polymorphic expressions of states 2 and 4 are scored as state 3; polymorphic expressions of states 4 and 6 are scored as state 5; polymorphic expressions of states 6 and 8 are scored as state 7.

149. M2 stylocone. The stylocone is an accessory cusp located along the upper molar stylar region, labial to the primary buccal cusps. Among afrotherians it is either present (state 1) or absent (state 0).

150. Expression of buccal cingula on M1-2 (MSO). Among afrotherians the labial cusps of the upper molars are either surrounded buccally by an extensive stylar shelf (state 0), a less well-developed “shelf” (2), a well-developed and continuous buccal cingulum (state 4), or a buccal cingulum that is poorly-developed, discontinuous, or absent (state 6). Polymorphic expression of states 0 and 2 is scored as state 1; polymorphic expression of states 2 and 4 is scored as state 3; polymorphic expressions of states 4 and 6 are scored as state 5.

151. Morphology of buccal cingulum around base of mesostyle on M1-2. Among Paleogene hyracoids (all of which have mesostyles), the buccal cingulum either traverses directly across the base of the mesostyle (state 0), or the cingulum ascends the mesostyle to form a triangular “peaked” cingulum in this region (state 2). Polymorphic expressions of states 0 and 2 are scored as state 1.

152. Buccal “ribs” on M1-2 ectoloph/centrocrista. Species of Saghatherium exhibit a peculiar condition in which the ectoloph is swollen labially and lingually, forming “ribs” between the major cusps (state 0). These ribs are not present in any other afrotherians (state 1).

153. Shape of ectoloph/centrocrista on M1-2 (MSO). Among afrotherians there is either no development of mesial (preparacrista), buccal (postpara- and premetacristae, together forming the centrocrista), and distal (postmetacrista) crests connecting the primary buccal cusps (state 0); alternatively these crests are present and are oriented roughly in line with the long axis of the toothrow (state 1), or the crests form strong angles relative to each other so that they form a “W”, as in the dilambdodont condition (state 2).

154. Upper molar protocone size on M1-2. Contains information similar to that of Asher's [2] character 42. Among afrotherians, the protocone is either equal in size to, or only slightly smaller than, the paracone (state 0), or the protocone is greatly reduced or absent (state 1).

155. Convergence of buccal and lingual cusps on M1-2 (MSO). The paracone and protocone are either widely separated and peripherally placed on the occlusal surface of the upper molars (state 0); moderately convergent, with the buccal cusps internalized relative to the buccal margin of the tooth (state 2); or the buccal and lingual cusps are strongly convergent, in some cases approaching each other's apices (state 4). Polymorphic expression of states 0 and 2 is scored as state 1; polymorphic expression of states 2 and 4 is scored as state 3.

156. Relative hypocone size on M1 (MSO). Contains information similar to that of Asher's [2] character 43. The hypocone is a distolingual cusp that is variably present on the upper molars of afrotherians. The cusp is either absent (state 0); present and distinct but smaller than the protocone (state 2); or present and approximately equal in size to, or larger than, the protocone (state 4). Polymorphic expression of states 0 and 2 is scored as state 1; polymorphic expression of states 2 and 4 is scored as state 3.

157. Mesostyles on M1-2. The mesostyle is an accessory fold of enamel that is situated on the buccal face of the ectoloph or centrocrista between the paracone and the metacone. It is either present (state 1), or absent (state 0).

158. Metacone on M1-2 (MSO). Contains information similar to that of Asher's [2] character 41. The metacone is either present and well-developed on the upper first and second molars (state 0), present but very small and “vestigial” (state 1, as in Potamogale), or absent (state 2, as in all technically “zalambdodont” afrotherians).

159. M1-2 paraconules (MSO). The paraconule is a small accessory cusp that is situated between the paracone and protocone of the upper molars. The M1-2 paraconules can either be present and well-developed (state 0); or there can be a paraconular “swelling” (not a distinct cusp) along the preprotocrista (state 1); the paraconule can also be either small and poorly developed (state 2) or absent (state 3).

160. M1-2 postparaconule crista. The postparaconule crista is a crest that courses distally or distolabially from the paraconule. It is either absent (state 0) or present (state 1).

161. M1-2 metaconules (MSO). The metaconule is a small accessory cusp that is situated between the metacone and protocone (or between the metacone and hypocone) of the upper molars. It is either present and well-developed (state 0), small and poorly developed (state 2), or absent/indistinct (state 4). Polymorphic expression of states 0 and 2 is scored as state 1; polymorphic expression of states 2 and 4 is scored as state 3.

162. Labial bulge of protocone on M1-2 (MSO). The M1-2 protocone of some Paleogene hyracoids is swollen labially, so that the cusp bulges into the lingual face of the paracone (state 2). Other afrotherians lack this feature (state 0). Polymorphic expressions of states 0 and 2 are scored as state 1.

163. Buccal cleft on M1-2. Asher's [2] character 50. The buccal margin of the upper molars is either flat or convex (state 0), or there is a distinct cleft in the buccal margin. This cleft is present between the paracone and metacone in those taxa that retain both cusps; in zalambdodonts the cleft is placed buccal to the dominant paracone (state 2). Polymorphic expressions of states 0 and 2 are scored as state 1.

164. Shape of M2 (MSO). The maximum mesiodistal length of the upper second molar is either less than 0.8 times the maximum buccolingual width of the tooth (state 0); between 0.81 and 1.2 times the maximum buccolingual width of the tooth (state 2); or longer than 1.21 times the maximum buccolingual width of the tooth (state 4). Polymorphic expression of states 0 and 2 is scored as state 1; polymorphic expression of states 2 and 4 is scored as state 3.

165. M1-2 parastyles (MSO). Many afrotherians have no parastyles on the upper first and second molars (state 0); if present, molar parastyles are either small (state 2) or large and well-defined (state 4). Polymorphic expression of states 0 and 2 is scored as state 1; polymorphic expression of states 2 and 4 is scored as state 3.

166. Morphology of M1-2 parastyles (MSO). When present, parastyles on the upper first and second molar are either hook-like and curve around the postmetacrista of the adjacent tooth (state 0); are directed mesially but are not hook-like (state 2); are weakly buccally oriented (state 4); or are strongly buccally oriented and buccally concave (state 6). Polymorphic expression of states 0 and 2 is scored as state 1; polymorphic expression of states 2 and 4 is scored as state 3; polymorphic expression of states 4 and 6 is scored as state 5.

167. Molar postmetacristae on M1-2. The postmetacrista courses distally from the apex of the metacone on upper molars; these crests are either short and directed buccally (state 0), long and buccally concave (state 1), long and well-developed but not buccally concave (state 3), or very weak or absent (state 4). Polymorphic expression of states 1 and 3 is scored as state 2.

168. Size of M2 relative to M1 (MSO). The upper second molar is either markedly larger than the first upper molar (state 0), subequal in size to the first upper molar (state 2), or is smaller than the first upper molar (state 4). Polymorphic expressions of states 0 and 2 are scored as state 1; polymorphic expressions of states 2 and 4 are scored as state 3.

169. Size of M3 relative to M2. The upper third molar is either markedly larger than the second upper molar (state 6), subequal in size to the second upper molar (state 4), or markedly smaller than the second upper molar (state 2). In some taxa the upper third molar is absent (state 0). Polymorphic expression of states 0 and 2 is scored as state 1; polymorphic expression of states 2 and 4 is scored as state 3; polymorphic expression of states 4 and 6 is scored as state 5.

170. Morphology of distal aspect of M3. There is considerable variation in the structure of the distal aspect of M3 among afrotherians. Some taxa have a hypocone on M3 from which a trenchant posthypocrista courses distally and buccally to form a low distocrista (state 0); in some taxa this distocrista is very tall and forms a high wall that encloses the distal aspect of the tooth (state 1). The Paleogene hyracoid Seggeurius exhibits a peculiar condition in which the posthypocrista curves around the metacone to meet the mesostyle (state 2). Other taxa have a hypocone but no posthypocrista (state 3), and some taxa lack an M3 hypocone (state 4). Polymorphic expression of states 0 and 3 is scored as state 5.

171. Interlocking of mesial and distal walls of upper molars (MSO). Some afrotherians have upper molars whose mesial margins are concave and cup the convex distal margin of the mesially adjacent tooth (state 2). Most taxa do not exhibit this interlocking condition (state 0). Polymorphic expressions of states 0 and 2 are scored as state 1.

172. Embrasure pits between upper molar protocones. In some afrotherians there are shallow to deep concavities in the alveolar bone of the maxilla, situated between the molar protocones, presumably to accommodate a tall trigonid of the lower molar during occlusion (state 2). Most taxa lack these pits (state 0). Polymorphic expressions of states 0 and 2 are scored as state 1.

Postcranium (173-271)

173. Clavicle. Contains information similar to that of Court's [20] character 45. The clavicle is either present (state 0) or absent (state 1).

174. Shape of scapular glenoid fossa. Contains information similar to that of Court's [21] character 35. When viewed ventrally, the scapular glenoid fossa is either oval in shape (state 0) or round (state 2). Polymorphic expressions of states 0 and 2 are scored as state 1.

175. Shape of cranial aspect of glenoid fossa (MSO). Contains information similar to that of Court's [21] character 36. The cranial aspect of the glenoid fossa is either flattened (state 0), shows moderate ventral expansion (state 1), or forms a massive hook-like structure (state 2).

176. Morphology of scapular coracoid process. The coracoid process is either absent or does not form an elongate projection from the supraglenoid region (state 0), or is elongate and medially inflected (state 1).

177. Metacromion. Contains information similar to that of Asher's [2] character 56, Frost et al.'s [1] character 71, and Court's [21] character 38. The metacromion process of the scapula is either present (state 0) or greatly reduced or absent (state 1).

178. Acromion process. The acromion process is either present and well-developed (state 0), or greatly reduced or absent (state 1).

179. Relative size of infra- and supraspinous fossae (MSO). Contains information similar to that of Court's [21] character 37. The infraspinous fossa is either markedly larger than the supraspinous fossa (state 2), markedly smaller than the supraspinous fossa (state 0), or approximately equal in size to the supraspinous fossa (state 1).

180. Shape of cranial margin of scapula. The cranial margin of the scapula is either distinctly arched (state 0), or it is flattened, with no arching of the border (state 1).

181. Shape of spinous process on axis (MSO). The spinous process of the axis is either large and "sickle-shaped", with processes extending cranially and caudally from the main body (state 0), small and sickle-shaped, with the cranial and caudal processes reduced (state 1), or it can take the form of more caudal spinous processes in the cervical vertebral column (state 2).

182. Total number of rib-bearing (thoracic) vertebrae (MSO). Taxa considered in this analysis have either 11 (state 0), 12 (state 1), 13 (state 2), 14 (state 3), 15 (state 4), 16 (state 5), 17 (state 6), 18 (state 7), 19 (state 8), or 20 or more (state A). I have scored Moeritherium as state A (20 or more thoracic vertebrae) following Andrews [22], who noted that 19 thoracic vertebrae had been recovered from an associated Moeritherium skeleton, and that at least one was missing. Polymorphic expressions of states 8 and A are scored as state 9.

183. Rib cross-section. All known living and extinct sirenians have dense, swollen ribs that are approximately circular in cross-section (state 1). All other taxa examined have relatively lightly constructed, flattened ribs (state 0).

184. Total number of lumbar vertebrae (MSO). Taxa considered in this analysis either have 3 (state 0), 4 (state 2), 5 (state 4), 6 (state 6), 7 (state 8), or 8 (state A) vertebrae between the rib-bearing thoracics and the first sacral vertebrae. Polymorphic expressions of these states are scored as 1, 3, 5, 7, and 9, respectively.

185. Orientation of spinous processes on lumbar vertebrae (MSO). Among afrotherians and other placental taxa the spinous processes of the lumbar vertebrae are either cranially oriented (state 0), oriented directly dorsally (state 1), or oriented caudally (state 2).

186. Craniocaudal orientation of lumbar transverse processes (MSO). In most taxa the transverse processes of the lumbar vertebrae are oriented transversely (state 2); in other taxa the transverse processes are either cranially oriented (state 0) or caudally oriented (state 4). Polymorphic expressions of states 0 and 2 are scored as state 1; polymorphic expressions of states 2 and 4 are scored as state 3.

187. Dorsoventral orientation of the lumbar transverse processes. In most taxa the transverse processes are oriented transversely (state 0); in some taxa the transverse processes are oriented ventrally (state 1).

188. Number of sacral vertebrae (MSO). Among afrotherians the number of sacral vertebrae varies between 0 and 7; this variation is delineated as 2 or fewer sacral vertebrae (state 0), 3 (state 2), 4 (state 4), 5 (state 6), 6 (state 8), 7 (state A), or 8 or more (state G). Polymorphic expressions of adjacent states along this morphocline are scored as 1, 3, 5, 7, 9, and C.

189. Number of vertebrae contributing to sacroiliac articulation (MSO). Asher's [2] character 64. Ingroup taxa vary from having only the first sacral vertebra contributing to the sacroiliac articulation (state 0), to having s1 and s2 contributing (state 2), to having s1 through s3 contributing (state 4), to having four or more sacral vertebrae contributing (state 6). Polymorphic expression of states 0 and 2 is scored as state 1; polymorphic expression of states 2 and 4 is scored as state 3; polymorphic expression of states 4 and 6 is scored as state 5.

190. Fusion of spinous processes on last two sacral vertebrae (MSO). A number of afrotherians exhibit fusion of the spinous processes on the last two sacral vertebrae (state 2). Many other ingroup taxa lack this feature (state 0). Polymorphic expressions of states 0 and 2 are scored as state 1.

191. Projection of the greater tuberosity of the humerus relative to the humeral head. Contains information similar to that of MacPhee's [23] character 20. Among afrotherians the greater tuberosity of the humerus either sits below, or at the level of, the proximal curvature of the humeral head (state 0), or it projects proximally well above the humeral head (state 1).

192. Shape of the deltoid tuberosity. Among afrotherians the deltoid tuberosity varies from being well-developed and laterally flared (state 0), to being less well-developed and forming a distinct ridge (state 1).

193. Length of the humeral medial epicondyle relative to trochlear width (MSO). Among afrotherians the medial epicondyle of the humerus is either absent or greatly reduced, being shorter than the mediolateral length of the humeral trochlea (state 0), or it is approximately equal in length to the mediolateral width of the humeral trochlea (state 1), or it is considerably longer than the mediolateral width of the humeral trochlea (state 2).

194. Position of lateral humeral epicondyle. Most taxa have a humeral lateral epicondyle that is placed either directly lateral, or only slightly proximal, to the distal humeral articulation (state 0). A number of proboscideans have a lateral humeral epicondyle that is shifted far proximally relative to the distal articular surface (state 1).

195. Placement of capitular tail. In those taxa with a bony capitular tail on the lateral aspect of the humeral capitulum, the structure is either placed at the distal aspect, or just at the proximodistal midpoint, of the capitulum (state 0) or it emerges from the capitulum far proximally (state 1). Some taxa lack a capitular tail (state 2).

196. Entepicondylar foramen. Court's [21] character 39. The entepicondylar foramen is a conduit on the medial aspect of the distal humerus that serves as a passageway for the median nerve and brachial artery. It is either present (state 0) or absent (state 2). Polymorphic expressions of states 0 and 2 are scored as state 1.

197. Entepicondylar foramen position relative to humeral trochlea. Among afrotherians the entepicondylar foramen is either placed relatively lateral, with the medial strut enclosing the foramen being confluent with the medial rim of the humeral trochlea (state 0), or the foramen is placed more medial, and the distal termination of the medial strut is offset medially with respect to the medial rim of the trochlea (state 1).

198. Orientation of humeral shaft with respect to the long axis of the radioulnar articulation. Among afrotherians the humeral shaft is either oriented approximately perpendicular to the long axis of the radioulnar articulation (state 0) or it is offset medially with respect to the long axis of the radioulnar articulation (state 1).

199. Capitular shape. Among the taxa analyzed in this study, the humeral capitulum varies from being globular and separated from the trochlear articulation by a distinct zona conoidea (state 0), to being confluent with the trochlear articulation, with no development of a zona conoidea (state 1), to being separated from the trochlea by a deep incision, but no flattened zona conoidea (state 2).

200. Relative proximodistal height of the humeral capitulum (MSO). Among the taxa considered in this study, the proximodistal height of the humeral capitulum is either less than 1.5 times the mediolateral width of the capitulum (state 0), between 1.51 and 2.0 times taller than its width (state 2), or over 2 times taller than its width (state 4). Polymorphic expression of states 0 and 2 is scored as state 1; polymorphic expression of states 2 and 4 is scored as state 3.

201. Dorsoventral depth of humeral condyles. The ventral face of the trochlear surface either does not project far beyond the ventral face of the distal humeral shaft (state 0), or the trochlear surface bulges ventrally, far beyond the ventral face of the shaft (state 1).

202. Morphology of olecranon fossa (MSO). Among afrotherians the olecranon fossa of the distal humerus is either shallow and only moderately concave (state 0), moderately recessed with medial and lateral walls that are well-defined (state 1), or deeply recessed (state 2).

203. Supracondylar foramen (MSO). In some afrotherians the bone above the distal humeral articulation is perforated through the olecranon fossa, leaving a large supracondylar foramen (state 2). Most taxa lack such a foramen (state 0). Polymorphic expressions of states 0 and 2 are scored as state 1.

204. Accessory elongate bone in arm. Contains information from Asher's [2] character 60. Chrysochlorids have a peculiar "third bone" in the forearm (state 1) that appears to be an ossified tendon of the flexor digitorum profundus muscle [24, 25]. All other taxa considered here lack such a third bone (state 0).

205. Orientation of olecranon process. Contains information similar to that of Court's [21] character 42. Among ingroup taxa the olecranon process is either oriented vertically (i.e., approximately parallel to the long axis of the ulnar shaft) (state 0), posteriorly deflected with respect to the long axis of the ulnar shaft (state 1), or medially deflected with respect to the long axis of the ulnar shaft (state 2).

206. Ulnar capitular facet. Some derived proboscideans have a large articular facet for the capitulum on the ulna (state 1). Most afrotherians lack such a facet (state 0).

207. Fusion of the radius and ulna. The radius and ulna are either fused (state 2) or unfused (state 0). Polymorphic expressions of states 0 and 2 are scored as state 1.

208. Ulnocarpal articulation. Contains information similar to that of Asher's [2] character 60 and Court's [21] character 45. The distal ulna either has a mediolaterally extensive articulation with the cuneiform and, in some cases, the pisiform (state 1) or the ulnar articulation is restricted to an elongate process projecting from the distal end of the shaft to provide a limited articulation with the carpus (state 0). In some taxa the ulna has an extensive articulation with the carpus, but the lateral aspect of the bone tapers into a small styloid process (state 2).

209. Cross-sectional shape of the ulnar shaft. Contains information similar to that of Court's [21] character 43. At midshaft the ulna is either transversely compressed (state 0), triangular, with three distinct ridges defined along its length (state 1), subcircular (state 2), or deeply excavated, as in macroscelidines (state 3).

210. Orientation of distal ulnar-carpal articulation with respect to the humeral articulation (Court's [21] character 44). The ulnar-carpal articulation is either semi-supinated (state 0) – that is, oriented roughly perpendicular to the long axis of the joint between the humerus and the radius/ulna, or pronated (state 1) with respect to the humeral articulation.

211. Ulnar-lunar articulation. Most afrotherians lack an articulation between the ulna and the lunar (state 0), but Arsinoitherium and various undoubted basal proboscideans have a distal ulnar articulation that overlaps the lunar (state 1).

212. Os centrale. Contains information similar to that of Court's [21] character 49. Among ingroup taxa the os centrale is either an isolated ossification in the distal carpal row (state 0) or it is fused with another carpal bone (state 1).

213. Lateral process of the cuneiform. A number of placentals have cuneiforms that have long laterally projecting hook-like processes emerging from the main body of the bone (state 1); in other taxa this carpal is blunt or rounded off laterally, with no development of such a hook-like process (state 0).

214. Shape of cuneiform. Among afrotherians the cuneiform is either distinctly wider mediolaterally than proximodistally tall (mediolateral width being gauged along the length of the unciform facet, height being gauged along extensor face) (state 0), approximately as wide mediolaterally as proximodistally tall (state 1), or much taller proximodistally than mediolaterally wide (state 2).

215. Morphology of external cuneiform-unciform articulation. Some ingroup taxa have lateral processes of the cuneiform that articulate distally in a distinct "stop-facet" on the lateral aspect of the unciform (state 0). Most taxa lack this lateral facet on the unciform (state 1).

216. Cuneiform-MCV articulation. In a few afrotherian taxa the cuneiform articulates with the fifth metacarpal (state 0). In most taxa the fifth metacarpal articulates exclusively with the unciform (state 1).

217. Unciform-MCIII contact. The unciform of most afrotherians has an internal articular surface for the third metacarpal (state 0), but a few taxa lack any contact between these bones (state 1).

218. Lunar-unciform contact (MSO). Contains information similar to that of Court's [21] character 46. Among afrotherians the lunar and unciform either do not contact (state 4), have a thin contact (as in Antilohyrax, state 2), or have extensive contact (state 0). Polymorphic expression of states 0 and 2 is scored as state 1; polymorphic expression of states 2 and 4 is scored as state 3.

219. Shape of anterolateral margin of unciform (MSO). Contains information similar to that of Court's [21] character 50. Afrotherians vary from having an anterolateral margin of the unciform that is either convex and overlapped by the cuneiform (state 2), a weak ridge separating the extensor margin of the unciform from the cuneiform articulation (state 1), or a distinctly upturned crest that projects proximally and buttresses the cuneiform (state 0).

220. Morphology of extensor margin of scaphoid. The extensor margin of the scaphoid either forms a distinct non-articular "block" in the proximal carpal row (state 0), or the extensor margin is narrow and thins to a narrow strip of exposed bone at the base of a convex articulation for the radius (state 1).

221. Presence of trapezium. Among afrotherians the trapezium is either present (state 0) or absent (state 1).

222. Morphology of scapho-radial articulation. The radial articulation of the scaphoid is either concave (state 0), flat or slightly convex (state 1), or concavoconvex (state 2).

223. Hinge joint between distal radius and scaphoid. Chrysochlorids have a distinctive hinge joint between the distal radius and the scaphoid (state 1). Almost all other afrotherians lack such a scapho-radial joint (state 0).

224. Pollex presence. The pollex is either present (state 0) or greatly reduced (vestigial) or absent (state 1).

225. Epipubic bones. Epipubic bones have been documented in marsupials and at least some stem placentals within crown Theria (state 0). All known crown placentals lack epipubic bones (state 1).

226. Cranial extent of ilia (Asher's [2] character 66). The iliac alae either extend cranially beyond the most cranial limit of the sacrum (state 0), or the cranial margins of the alae terminate near the cranial margin of the sacrum (state 1).

227. Anterior inferior iliac spine. Aardvarks and macroscelideans have a distinct tubercle situated cranial to the acetabulum (state 1). Most other taxa lack such a tubercle (state 0).

228. Iliopectineal tubercle (Asher's [2] character 68). Some afrotherians have a distinct iliopectineal tubercle placed inferior to the acetabulum (state 1). Other taxa do not have such a tubercle (state 0). 

229. Shape of iliac alae. Contains information similar to that of Court's [21] character 57. The iliac alae are either narrow, with no mediolateral or dorsoventral flare (state 0), or wide, flaring, and obliquely aligned with respect to the mediolateral plane (state 1).

230. Size of obturator foramen (MSO). Among afrotherians the obturator foramen varies in size from being as large, or larger than, the acetabulum (state 0), to being smaller than the acetabulum or absent (state 2). Polymorphic expressions of states 0 and 2 are scored as state 1.

231. Accessory foramen cranial to obturator foramen (MSO). Some macroscelideans have an accessory foramen placed cranial to the obturator foramen (state 2). All other taxa lack this accessory foramen (state 0). Polymorphic expressions of states 0 and 2 are scored as state 1.

232. Morphology of pubic symphysis (MSO). Contains information similar to that of Asher's [21] character 62 and MacPhee's [23] character 22. Among afrotherians the pubic symphysis varies from being present and craniocaudally extensive (state 0), to being present but restricted (state 2). In some taxa the pubic symphysis is absent, either with midline contact of the pubes (state 4) or with widely separate pubes (state 6). Polymorphic expression of states 0 and 2 is scored as state 1; polymorphic expression of states 2 and 4 is scored as state 3; polymorphic expression of states 4 and 6 is scored as state 5.

233. Mediolateral orientation of superior pubic ramus (MSO). The superior pubic rami are either oriented transversely (state 0), are "v"-shaped, meeting each other ventrally at a sharp angle (state 2), or are oriented almost directly ventrally, leaving a wide midline gap (state 4). This character is considered to be independent of character 223 because taxa can have, e.g., superior pubic rami that are ventrally oriented or "v"-shaped but nevertheless have a craniocaudally elongate pubic symphysis. Polymorphic expression of states 0 and 2 is scored as state 1; polymorphic expression of states 2 and 4 is scored as state 3.

234. Orientation of superior pubic ramus with respect to the ventral face of the ilium. Contains information similar to that of Asher's [2] character 70. The superior pubic ramus either meets the ilium at an angle (at least 15 degrees relative to the ischium) (state 1) or is oriented roughly in line with the ventral face of the ilium (state 0).

235. Orientation of inferior ischiopubic rami. The inferior ischiopubic rami are either oriented medially and cranially (state 0) or transversely or caudally (state 1).

236. Projection of greater trochanter of femur. Some afrotherians have femoral heads that project above the greater trochanter (state 1); in other taxa the femoral head is either situated at the level of, or below, the greater trochanter (state 0).

237. Morphology of fovea capitis. Contains information similar to that of MacPhee's [23] character 27 and Court's [21] character 55. The fovea capitis (the site of attachment for the ligamentum teres) is either a discrete pit that is placed centrally on the femoral head (state 0), ventrally open, so that the femoral head is grooved (state 1), or is absent or poorly developed (state 2).

238. Shape of lesser trochanter. Among afrotherians the lesser trochanter varies from being a bulbous tuberosity (state 0), to being an elongated flange (state 1), to being greatly reduced in the form of a low rugosity (state 2).

239. Intertrochanteric crest. In some taxa a crest descends from the lesser and/or greater trochanter to form an intertrochanteric crest (state 0); in other taxa there is no distinct crest projecting toward the midline from these trochanters (state 1).

240. Femoral neck. Some ingroup taxa have a distinct femoral head that is offset from the femoral shaft by a thin femoral neck that is much narrower than the maximum diameter of the femoral head (state 1). In other taxa there is no marked constriction distal to the femoral head (state 0).

241. Gluteal tuberosity. Among afrotherians the gluteal tuberosity is either absent (state 0), present in the form of a bulbous tuberosity (state 1), present in the form of an elongate plate or flange (state 2), or present in the form of a low rugosity (state 3).

242. Position of gluteal tuberosity. The gluteal tuberosity is either placed near midshaft (state 0), or more proximal, near the position of, or just distal to, the lesser trochanter (state 1).

243. Femoral condyle asymmetry. Contains information similar to that of Court's [21] character 55. Many afrotherians have femoral condyles that are roughly symmetrical in size and shape (state 0), while others have condyles that exhibit clear asymmetry in size and/or shape (state 1).

244. Shape of patellar groove. The patellar groove is either proximodistally reduced and mediolaterally as wide as, or slightly wider than, its proximodistal height when viewed ventrally (state 0), or it is proximodistally elongate, being taller than wide (state 1).

245. Falciform process on tibia. Some afrotherians have a bulbous or blade-like falciform process emerging from the anterior and proximal aspect of the tibial shaft (state 1). Other taxa have no such process, and the anterior aspect of the proximal tibia is blunt or has only a low rugosity (state 0).

246. Proximal tibiofibular fusion. The tibia and fibula are either solidly fused proximally (state 1) or are unfused (state 0).

247. Distal tibiofibular fusion (MSO). The tibia and fibula are either solidly fused distally (state 2) or are unfused (state 0). Polymorphic expressions of states 0 and 2 are scored as state 1.

248. "Beaked" distal tibial articulation. Some afrotherians have a distal tibial articulation with the astragalus whose anterior border is distinctly "v"-shaped in ventral view due to the development of a distally protruding ridge that articulates in a deep incision along the midline of the astragalar trochlea (state 1). In other taxa the anterior border of the distal tibial articulation is flat (state 0).

249. Accessory tubercles on proximal fibula. Some afrotherians have accessory tubercles or flanges along the length of the proximal fibula (state 1). The proximal fibular shaft of most other taxa is smooth, with no clear development of these tubercles (state 0).

250. Size and shape of the medial malleolus of tibia. Contains information similar to that of Court's [21] character 58. In taxa whose tibial medial malleolus is large and well-developed, the articular surface for the astragalus is either oriented at least in part anteriorly (state 0) or directly medially (state 1). Some taxa have no medial malleolus or only a small, plate-like, malleolus (state 2).

251. Astragalar-cuboid contact. Among afrotherians contact between the astragalar head and cuboid is either absent or negligible (state 0), or present and well-developed (state 1).

252. Elevation of the lateral trochlear rim of the astragalus. Many taxa have medial and lateral trochlear margins that are subequal in height (state 0). In some afrotherians the lateral trochlear rim is clearly elevated relative to the medial margin (state 1).

253. Morphology of junction between lateral rim of astragalar trochlea and fibular facet. Some stem placentals (e.g., Asioryctes) and many marsupials have poorly developed fibular malleoli that do not form a stable interlocking articulation with the lateral border of the astragalar trochlea (state 1). All of the placentals examined in this study have a sharply defined lateral rim of the astragalar trochlea (state 0).

254. Relative size of astragalar ectal and sustentacular facets (MSO). Contains information similar to that of Court's [21] character 62. Either the ectal and sustentacular facets of the astragalus are approximately equal in size (state 2), or the ectal facet is distinctly smaller than (state 4) or larger than (state 0) the sustentacular facet. Polymorphic expression of states 0 and 2 is scored as state 1; polymorphic expression of states 2 and 4 is scored as state 3.

255. Shape of astragalar ectal facet. Among afrotherians the ectal facet either takes a triangular (state 1) or rectangular (state 0) shape. All known living and extinct hyracoids have confluent ectal and sustentacular facets (state 2). In some mammals the ectal facet is reduced to a thin strip (state 3).

256. Position of sustentacular facet on astragalus (Court's [21] character 61). In some afrotherians the sustentacular facet is either oriented toward, or joins, the medial side of the astragalar head (state 0); in other taxa the sustentacular facet is oriented toward, or joins, the lateral side of the astragalar head (state 1).

257. Proximal path of sustentacular facet. The sustentacular facet either does (state 1) or does not (state 0) ascend the medial tubercle buttressing the groove for flexor fibularis.

258. Position of calcaneus relative to astragalus. In crown placentals the astragalus is superposed above the calcaneus (state 0); in more distantly related taxa it is only partially superposed or placed medial to the calcaneus (state 1).

259. Cotylar fossa. Contains information similar to that of MacPhee's [23] character 28. Most placentals lack a distinct cotylar fossa for articulation with the medial malleolus of the tibia (state 0). Among those taxa that have a cotylar fossa, it can be open plantarly (state 1), or enclosed plantarly (state 2).

260. Astragalar tuberculum mediale (=posteromedial process). Contains information similar to that of MacPhee's [23] character 29 and Court's [21] character 64. Some afrotherians have a distinct tubercle projecting medially from the proximal trochlear articular surface (state 1); in other taxa this tubercle is absent or poorly developed (state 0).

261. Entocuneiform-navicular buttressing. In some afrotherians the proximal aspect of the entocuneiform buttresses and obscures much of the navicular in plantar view (state 0). In other taxa the entocuneiform and navicular have a dorsoplantarly oriented articulation and the entocuneiform provides no plantar buttressing to the navicular (state 1).

262. Navicular facet of the astragalus. The navicular facet of the astragalus is either distally flattened (state 1) or convex (state 0). Antilohyrax and Titanohyrax are unique in having a concavoconvex navicular facet (state 2).

263. Morphology of astragalar neck/astragalar body connection. In many placentals the lateral border of the astragalar neck meets the astragalar body near its midline, so that the neck is medially "offset" from the body (using the terminology of Pickford et al. [26] (state 0). In other taxa the lateral border of the astragalar neck meets the astragalar body at or near the lateral trochlear rim (state 1).

264. Distal process of the navicular. Some afrotherians have a long process that projects distally from the plantar and distal aspect of the navicular body (state 1). Other taxa have no such process on the navicular (state 0).

265. Peroneal tubercle (MSO). Contains information similar to that of Asher's [2] character 71. Tenrecs and golden moles have prominent peroneal tubercles that project distally from the lateral body of the calcaneus (state 4). In other taxa the peroneal tubercle may be present but relatively small and not distally projecting (state 2), or absent (state 0). Polymorphic expression of states 0 and 2 is scored as state 1; polymorphic expression of states 2 and 4 is scored as state 3.

266. Shape of cuboid facet on the calcaneum. The facet on the calcaneum for articulation with the cuboid is either roughly circular in shape (state 0), takes the shape of a transversely oriented oval (state 1), takes the shape of a vertically oriented oval (state 2), or takes the shape of a transversely oriented arc (state 3).

267. Naviculocalcaneal facet. An articular facet between the navicular and the calcaneus is either present (state 1) or absent (state 0).

268. Size of calcaneal plantar tubercle (Court's [21] character 66). Proboscideans have a massive tubercle on the plantar surface of the calcaneus (state 1); other afrotherians have little or no development of the calcaneal plantar tubercle (state 0).

269. Calcaneal-fibular facet. Contains information from Asher's [2] character 71. A number of afrotherians have an articulation between the calcaneus and the fibula (state 0); in other taxa the fibula does not extend to the calcaneus, and there is no articulation between the two bones (state 1).

270. Hallux. The hallux is either present (state 0) or absent (state 1).

271. Pedal digit V. The fifth pedal digit is either present (state 0) or absent (state 1).

Mandibular characters (272-289)

272. Morphology of mandibular angle (MSO). Among some afrotherians the mandibular angle takes the shape of a thin, distally protruding process, tapering to a point distally and being either medially inflected (state 0) or in line with the horizontal ramus (state 1). In other taxa the angle does not taper to a point and is rounded off distally. Among these taxa, there is either a distally protruding, rounded process (state 2), or the distal aspect of the mandibular angle is completely rounded off, with no sign of a protruding process (state 3).

273. Distal terminus of the mandibular symphysis (MSO). Among afrotherians the posterior margin of the mandibular symphysis varies considerably in its placement relative to the toothrow. Ingroup variation can be accommodated by 7 states: the distal margin either extends to the mesial aspect, or along the length of, the first lower molar (state 0), p4 (state 2), p3 (state 4), p2 (state 6), p1 (state 8), the lower canine (state A), or the last lower incisor (state G). The proboscidean Phiomia lacks p2 but has a mandibular symphysis that terminates well mesial to the p3 and at the probable former position of the missing p2 (assuming the same position observable in more basal taxa such as Numidotherium or Moeritherium, which lack a diastema between p2 and p3). For this reason Phiomia was scored as exhibiting state 4, despite the fact that this taxon lacks the tooth described in the character state definition. Dasypus was assigned state 9 (despite the fact that this genus retains no lower incisors) because it is clear that the symphysis did not extend back to the lower canine or premolars. Didelphis and Orycteropus are scored as “missing” for this character; the former due to uncertainty surrounding the homology of its lower premolars, and the latter due to premolar loss. Taxa with horizontal replacement are scored as inapplicable for this character. Polymorphic expression of states 0 and 2 is scored as state 1; polymorphic expression of states 2 and 4 is scored as state 3; polymorphic expression of states 4 and 6 is scored as state 5; polymorphic expression of states 6 and 8 is scored as state 7; polymorphic expression of states 8 and A is scored as state 9; polymorphic expression of states A and G is scored as state C.

274. Mediolateral constriction of mandibular symphyseal region. Contains information similar to that of Domning's [4] character 121. Many living and extinct sirenians exhibit a unique configuration of the mandibular symphysis in which the rostral aspect of the lower jaw is mediolaterally constricted, with a distinct junction between the distal tooth row and a more tubular rostral region (state 1). Although some Paleogene hyracoids, such as Geniohyus, and some proboscideans, such as Barytherium and Palaeomastodon, exhibit some constriction of the symphyseal region with respect to the toothrow, the condition observable in these taxa and their close relatives is difficult to distinguish consistently and so is scored as being absent, as in most other afrotherians (state 0).

275. Shape of ventrorostral border of horizontal mandibular ramus. Contains information similar to that of Domning's [4] character 122. Some derived sirenians have a mandibular symphyseal region that is ventrally deflected with respect to the postcanine toothrow, leading to a distinctively downturned, and ventrally concave, appearance (state 1). All other known afrotherian taxa lack this distinctive feature (state 0).

276. Rapid deepening of the mandible below anterior premolars (MSO). Although many afrotherian taxa have relatively “deep” mandibles, the mandibular morphology of the Paleogene hyracoids Geniohyus and Bunohyrax major is distinctive in that the ventral border of the horizontal ramus exhibits a sharp transition from a relatively shallow symphyseal region, deepening rapidly to achieve maximum depth under the premolar region (state 2). This condition leads the ventral border of the horizontal ramus to be at least moderately concave, whereas all other taxa have flat or convex horizontal rami in this region (state 0). Polymorphic expressions of states 0 and 2 are scored as state 1.

277. Internal mandibular fenestra (MSO). One of the most enigmatic features of Paleogene hyracoids is the internal mandibular fenestra, a perforation in the lingual aspect of the horizontal mandibular ramus that is either situated, or first appears ontogenetically, under the posterior molars. In this study I consider this fenestra to be expressed in two different forms - either the fenestra is “small” (state 1), regardless of shape (the fenestra can be circular, oval, or triangular), or the fenestra is very large, being mesiodistally extensive and forming a large mandibular “fossa” (state 2). Although there seems to be considerable confusion in the literature concerning the origin of mandibular “fenestra” and “fossa”, I consider it likely that the “fossa” is simply the end result of extensive pneumatization from a small “fenestra”, and so I score this character as ordered. All other afrotherians lack this feature (state 0). As the presence or absence of this character may be due to sexual dimorphism, it is necessary to have adequate samples to determine whether or not it is truly absent; for this reason Seggeurius and Microhyrax are scored as “missing” due to small sample sizes. I have scored other, non-hyracoid taxa represented by small samples as “absent” simply because this feature has never been documented outside of the order Hyracoidea.

278. Pneumatization of mandible (MSO). Some Paleogene hyracoids further differ from all other afrotherians in having the mandible pneumatized to varying degrees from the internal mandibular fenestra. In some taxa, such as Pachyhyrax, an internal mandibular fenestra is present but only the horizontal ramus is pneumatized (state 1), whereas in certain species of Thyrohyrax both the horizontal and vertical mandibular rami are pneumatized (state 2). No other afrotherians are known to exhibit either of these patterns of pneumatization, and are assigned state 0.

279. Relative depth of horizontal mandibular ramus below m2 (MSO). Contains information similar to that of Domning's [4] character 128. The maximum depth of the horizontal mandibular ramus is expressed as a ratio, relative to the maximum width of the m2 trigonid - being either less than 2 times greater than the maximum width of the m2 trigonid (state 0), greater than 2 but less than 4 times (state 2), greater than 4 but less than 6 times (state 4), or greater than 6 times (state 6). Polymorphic expression of states 0 and 2 is scored as state 1; polymorphic expression of states 2 and 4 is scored as state 3; polymorphic expression of states 4 and 6 is scored as state 5.

280. Coronoid canal (MSO). The coronoid canal is a foramen at the base of the coronoid process, just behind m3, that is present in all living and extinct hyracoids for which this region is known (or well-preserved). The canal is scored as being either present (state 2) or absent (state 0). Polymorphic expressions of states 0 and 2 are scored as state 1.

281. Exposure of the dental capsule (MSO). Domning's [4] character 127. The mandibular dental capsule is either exposed along the medial side of the ascending ramus (state 2) or is not exposed or absent, being completely enclosed by bone (state 0). Polymorphic expressions of states 0 and 2 are scored as state 1.

282. Meckelian sulcus. Some stem placentals and stem therians retain a distinct Meckelian sulcus on the lingual aspect of the mandible (state 0). All crown placentals analyzed in this study lack this sulcus (state 1).

283. Height of coronoid process relative to mandibular condyle (MSO). Among ingroup taxa the coronoid process either sits at the level, or only just above, the mandibular condyle (state 0), or it projects slightly above the condyle (state 2), or it projects high above the condyle (state 4). Polymorphic expression of states 0 and 2 is scored as state 1; polymorphic expression of states 2 and 4 is scored as state 3.

284. Orientation of ascending ramus with respect to occlusal plane (MSO). Among afrotherians, the anterior border of the ascending mandibular ramus is either anteriorly inclined with respect to a plane passing perpendicular to the occlusal surface of the lower molars (state 4), vertically oriented (state 2), or posteriorly inclined (state 0). Polymorphic expressions of states 0 and 2 are scored as state 1; polymorphic expressions of states 2 and 4 are scored as state 3.

285. Origin of ascending ramus of mandible (MSO). The anterior border of the ascending ramus of the mandible either ascends far distal to the distal margin of the third lower molar (state 0), just at the distal margin the third lower molar (state 2), along the length of the third lower molar, but distal to the second lower molar (state 4), or the ascending ramus obscures the second lower molar (state 6). This character is only scored in taxa represented by individuals with fully erupted lower third molars. Polymorphic expression of states 0 and 2 is scored as state 1; polymorphic expression of states 2 and 4 is scored as state 3; polymorphic expression of states 4 and 6 is scored as state 5.

286. Height of mandibular condyle. Contains information similar to that of Novacek's [27] character 72. The mandibular condyle is placed either far above the occlusal plane of the lower molars (state 1), or is placed approximately at the level of the occlusal plane of the lower molars (state 0).

287. Mandibular condyle shape. Most afrotherians have a mandibular condyle that is transversely elongate (state 2). Extant Procavia and some Paleogene hyracoids exhibit a condition in which the condyle in transversely elongate, but the medial aspect of the condyle is rostrocaudally constricted so that the condyle thins markedly medially (state 3). Other afrotherians have either a featureless, plate-like mandibular condyle that is not expanded transversely (state 1), or a condyle that is ball-shaped (state 0).

288. Endocoronoid crest (MSO). In some afrotherians the medial face of the ascending mandibular ramus is buttressed by a variably developed crest that descends from the medial side of the mandibular condyle, trending rostroventrally to meet the base of the coronoid process (state 2). In other taxa this area is smooth, with no obvious crest present (state 0). Polymorphic expressions of states 0 and 2 are scored as state 1.

289. Excavation of medial and inferior aspect of the mandibular condyle. Procavia and some extinct hyracoids exhibit a deep pit under the medial aspect of the lateral condyle, presumably the area of insertion for the lateral pterygoid muscle (state 2). In other afrotherians this area is flat, which no clear excavation or fossa for this muscle insertion (state 0). Polymorphic expressions of states 0 and 2 are scored as state 1.

Cranial characters (290-385)

290. Papillary cartilage (Asher's [28] character 17). As discussed by Asher [28], some afrotherians exhibit a cartilaginous structure within the palatine papilla (the structure that is defined bilaterally by the nasopalatine ducts as they open into the oral cavity) (state 0); most placentals have no cartilage within the palatine papilla (state 2). Polymorphic expressions of states 0 and 2 are scored as state 1.

291. Vomeronasal organ blood vessels (Asher's [28] character 18). In many placentals “a prominent blood vessel travels anteroposteriorly along with the vomeronasal organ tucked into its lateral side, giving the vomeronasal organ a kidney-shaped appearance when viewed coronally” (Asher [28], p. 42) (state 0). In some afrotherians the vomeronasal organ has blood vessels scattered throughout its epithelium (state 1).

292. Oral opening of the nasopalatine duct (Asher's [28] character 12). Among taxa for which data are available, the nasopalatine duct (which provides a connection between the nasal fossa and the oral cavity via incisive foramina) is either paired and opens into the mouth on either side of the palatine papilla (state 0), or the duct opens into the oral region via a single, unpaired channel (state 1).

293. Connection of nasopalatine duct and paraseptal cartilages (Asher's [28] character 14). Among taxa for which data are available, the nasopalatine duct cartilage either appears adjacent to the nasopalatine duct, with no connection to the paraseptal cartilage (state 0), or the nasopalatine duct cartilage connects to the paraseptal cartilage posterior to the nasopalatine duct (state 1).

294. Paraseptal cartilage and anterior vomeronasal organ ("outer bar") (Asher's [28] character 11). Among afrotherians a strut of paraseptal cartilage either consistently encloses the vomeronasal organ anterolaterally (state 0), or the paraseptal cartilage is laterally open along its anterior half (state 2). Polymorphic expressions of states 0 and 2 are scored as state 1.

295. Nasopalatine duct cartilage (Asher's [28] character 13). As discussed by Asher [28], the nasopalatine duct cartilage is usually present along the medial and/or lateral side of the nasopalatine ducts (state 1); in some ingroup taxa this cartilage is reduced or absent (state 0).

296. Nasolacrimal duct covering (Asher's [28] character 16). Among afrotherians for which data are available, the nasolacrimal duct is either laterally shielded by cartilage (state 1) or laterally open (state 0) [28].

297. Shape of nasal septum (Asher's [28] character 19). As discussed by Asher [28] and Sánchez-Villagra [29], most placentals have a ventrally ovoid nasal septum (state 1). This condition differs from that observable in marsupials and some placentals, who have a parallel-sided nasal septum (state 0).

298. Configuration of incisive foramina (Court's [20] character 3). Therians generally have two paired incisive foramina offset laterally on either side of the midline palatal suture between the premaxillary bones (state 0). Proboscideans and sirenians exhibit a peculiar condition in which the incisive foramina are confluent, leading to the presence of a single foramen (state 1).

299. Shape of incisive foramina. Contains information similar to that of Novacek's [27] character 3. Among those placentals that have paired incisive foramina, these foramina are either oval and rostrocaudally elongate (state 0), subcircular with no rostrocaudal elongation (state 1), or are reduced to thin fissures (state 2).

300. Constriction of palate anterior to postcanine tooth sequence. Most placentals have palatal margins that arc gradually in a rostromedial direction toward the premaxillary region (state 0). In some afrotherians the maxillary palatal margins are tightly constricted just anterior to the most mesial premolar (or molar, in the case of adult Trichechus) (state 1).

301. Precanine fossa (MSO). Some ingroup taxa have a deep fossa on the lateral face of maxilla or premaxilla, just dorsal to the alveolar margin and mesial to the upper canine. These fossae often serve as channels that accommodate particularly enlarged lower canines as they occlude in front of the upper canine, but they are also present in some Paleogene hyracoids that lack enlarged lower canines. Although these different functional expressions strongly suggest that the feature is not homologous across all of the ingroup taxa that exhibit this character, its presence (state 2) is nevertheless potentially phylogenetically informative at lower levels among afrotherians, as most taxa lack a precanine fossa (state 0). Polymorphic expressions of states 0 and 2 are scored as state 1.

302. Common recess for sphenopalatine and dorsal palatine foramina (Novacek's [27] character 20; Asher's [2] character 21). In some afrotherians the sphenopalatine foramen, which transmits the sphenopalatine nerve, artery, and vein to the posterior nasal fossa, travels in a common recess (state 0) with the dorsal palatine foramen, which encloses the descending palatine artery and nerve traveling to palatal region [30]. Alternatively, these foramina can be offset from each other within the palatine bone, forming two distinct conduits (state 1).

303. Configuration of postpalatine region (MSO). Contains information similar to that of Novacek's [30] character 18 and Domning's [4] character 97. The distal margin of the palatine bones along the posterior edge of the hard palate varies considerably among afrotherians -- from being flat (state 1), to having a distinct spine projecting distally from the midline suture of the palatines (state 0), to having an incision projecting between mesially between the palatines (state 2).

304. Postpalatine torus (MSO). Contains information similar to that of Novacek's [30] character 19. Some afrotherians exhibit a distinct thickening of the distal margin of the palatine bones, along the posterior edge of the hard palate - here referred to as a “postpalatine torus” (state 0). Most afrotherians lack such a torus (state 2). Polymorphic expressions of states 0 and 2 are scored as state 1.

305. Rostral extension of midline palatal margin, relative to toothrow (MSO). Contains information similar to that of Novacek's [30] character 17. Among afrotherians the most mesial placement of the caudal border of the palatines along the midline varies from being situated along the length of, or mesial to, the third upper molar (state 0), to being situated at the distal edge of, or only slightly behind, the third upper molar (state 2), to being situated far behind the third upper molar (state 4). Taxa with horizontal replacement of teeth in the upper dentition (Loxodonta and Trichechus) are scored as "inapplicable" for this character due to ambiguity regarding tooth loci. Polymorphic expressions of states 0 and 2 are scored as state 1; Polymorphic expressions of states 2 and 4 are scored as state 3.

306. Lacrimal foramen presence/position. Contains information similar to that of Asher's [2] character 24, Novacek's [30] character 23, Court's [20] character 7, Domning's [4] character 91, and MacPhee's [23] character 5. A number of fully or semi-aquatic afrotherians lack a lacrimal foramen (state 0). In those taxa that retain a lacrimal foramen, it is either placed on the facial process of the lacrimal bone, widely exposed on the side of the face outside of the orbit (state 1); exposed on the rim of the orbit but buttressed rostrally by the orbital wing of the maxilla or an anterior lacrimal crest (state 2), or it is completely enclosed within, and opens directly into, the orbit (state 3). Scoring for Barytherium is based on Court [20], who noted that there is no lacrimal foramen present in the undescribed specimen of B. grave from Dor el Talha, Libya.

307. Lacrimal tubercle (MSO). Contains information similar to that of Novacek's [30] character 24. Living and extinct hyracoids are well-known for having a distally projecting tubercle on the lateral face of the lacrimal bone (state 2). Most afrotherians lack this tubercle (state 0). Polymorphic expressions of states 0 and 2 are scored as state 1.

308. Lacrimal-palatine contact. Contains information similar to that of Novacek's [30] character 21. Contact between the lacrimal and palatine bones within the orbital mosaic is either present (state 0) or absent (state 1) among afrotherians. I have chosen to assign taxa without lacrimal bones state 1 rather than “missing” or “inapplicable”.

309. Infraorbital fossa. Derived proboscideans exhibit a deep fossa inferior to the orbit and rostral branch of the zygomatic arch (state 1). A less pronounced, but superficially similar, fossa is present in the xenarthran Dasypus. All other ingroup taxa lack this fossa (state 0).

310. Length of infraorbital canal (MSO). Contains information similar to that of Asher's [2] character 29, Novacek's [30] character 10, Frost et al.'s [1] character 6, and Court's [20] character 4. A number of afrotherians have an infraorbital canal whose lateral enclosure has been reduced to a thin strut that is either the same length, or rostrocaudally shorter than, the mesiodistal length of the second upper molar (state 2). Most taxa have a much longer infraorbital canal (state 0). Polymorphic expressions of states 0 and 2 are scored as state 1.

311. Position of anterior opening of infraorbital canal (MSO). The anterior opening of the infraorbital canal, here determined from the most caudal point along the lateral wall delimiting the opening of the infraorbital canal, is either placed above, or rostral to, the second upper premolar (state 0), above the third upper premolar (state 2), above the fourth upper premolar (state 4), above the first upper molar (state 6), or above the second or third upper molar (state 8). Taxa with horizontal replacement of teeth in the upper dentition (Loxodonta and Trichechus) are scored as "inapplicable" for this character due to ambiguity concerning tooth loci. Polymorphic expressions of states 0 and 2 are scored as state 1; polymorphic expressions of states 2 and 4 are scored as state 3; polymorphic expressions of states 4 and 6 are scored as state 5; polymorphic expressions of states 6 and 8 are scored as state 7.

312. Morphology of the anterior opening of the infraorbital canal. In some afrotherians the maxilla just anterior to the medial wall of the infraorbital canal is deeply excavated, leaving a distinct impression in the form of a fossa or gutter (state 0). Most afrotherians lack this excavation (state 2). Polymorphic expressions of states 0 and 2 are scored as state 1.

313. Maxillary/jugal contribution to the anterior rim of the orbit (MSO). Contains information similar to that of Court's [20] character 11 and Domning's [4] character 85. The lateral rim of the orbit can be formed either completely by the maxilla (state 0), or the jugal can project rostrally from the zygomatic arch to provide a contribution that is roughly equal to that of the maxilla (state 1). In some taxa the jugal sends a long projection across the anterior orbital rim that approaches, or actually contacts, the lacrimal in the dorsomedial corner of the orbit (state 2).

314. Bulging of the premaxilla. Sirenians exhibit a derived condition in which the premaxillary symphysis is rostrocaudally extensive and the region as a whole is swollen and markedly convex in the rostrocaudal and mediolateral planes (state 1). In most other afrotherian taxa the premaxillary symphysis is short and is not so clearly swollen (state 0).

315. Shape of dorsocaudal aspect of premaxilla. Among afrotherians the dorsocaudal aspect of the premaxilla forms either a vertical, near-vertical, or rostrocaudally inclined articulation with the maxilla (state 0), or it tapers caudally to form a thin maxillary or frontal process (state 1).

316. Caudal extent of premaxillary retraction (MSO). The point of greatest premaxillary retraction is either insubstantial, being placed above the upper incisors (state 0), or the retraction can extend back above the upper canine (state 1). In some taxa premaxillary retraction is considerable, extending at least to the region above the third upper premolar (state 2). I have assigned Rhynchocyon and Dasypus, both of which lack upper incisors, state 0, because their premaxillae are not exposed above the upper canine.

317. Nasomaxillary fossa. Some Paleogene hyracoids have a peculiar fossa or foramen of unknown function that perforates the rostrum, evidently along the suture between the premaxilla and maxilla. Whitworth [31] has referred to this feature as being a “nasomaxillary fossa” (state 1). No other afrotherians exhibit this fossa (state 0).

318. Position of anterior rim of orbit (MSO). Among afrotherians the anterior rim of the orbit is either placed caudal to the upper molars (state 6), above the upper molars (state 4), above the upper premolars (state 2), or anterior to the upper premolars (state 0). Polymorphic expressions of states 0 and 2 are scored as state 1; polymorphic expressions of states 2 and 4 are scored as state 3; polymorphic expressions of states 4 and 6 are scored as state 5.

319. Rostral flaring of inferior orbital margin (MSO). In some proboscideans and sirenians the inferior orbital margin projects rostrally, imparting a “flared” appearance to the jugal or maxillary bone. In some taxa this flaring is situated well above the toothrow (state 1), but in derived sirenians the inferior orbital margin flares just above the alveolar margin (state 2). Most other afrotherians have an anterior orbital margin that exhibits no rostral flare (state 0). 

320. Premaxilla-frontal contact (MSO). Contains information similar to that of Novacek's [30] character 4, Court's [4] character 2, and Domning's [4] character 9. Among afrotherians a contact between the premaxilla and frontal is either present (state 2) or absent (state 0). Polymorphic expressions of states 0 and 2 are scored as state 1.

321. Maxilla-frontal contact. Contains information similar to that of Asher's [2] character 31 and Novacek's [30] character 6. Among afrotherians there is either little contact, or only a thin contact, between the maxilla and frontal along the side of the rostrum (state 0), or there is an extensive contact between these two bones (state 1).

322. Shape of nasals (MSO). Contains information similar to that of Asher's [2] character 32, Novacek's [30] character 2, and MacPhee's [23] character 6. The shape of the nasals varies among afrotherians from being essentially straight throughout their length (state 1), to narrowing caudally, so that the nasals together form an apex at the nasofrontal suture (state 0), to widening posteriorly (state 2). Those taxa whose nasals widen posteriorly and then taper caudally are assigned state 2.

323. Extent of rostral nasal projection relative to premaxilla (MSO). Contains information similar to that of Novacek's [30] character 1 and Domning's [4] character 8. Many Paleogene hyracoids have nasals that project anteriorly far beyond the most rostral point on the naso-premaxillary suture (state 0). Arsinoitherium is assigned the same state due to its greatly inflated nasal horn cores, which also project beyond the most rostral point on the naso-premaxillary suture. Some taxa have nasals whose rostral margin is essentially confluent with the rostral margin of the premaxilla (state 1), while others have nasals that are markedly retracted relative to the premaxilla (state 2).

324. Shape of dorsal aspect of nasal aperture, when viewed dorsally (MSO). The rostral nasal margin is either rostrally convex, projecting anteriorly beyond the most caudal border of the nasal aperture (state 0), flat or slightly concave (state 1), or deeply incised along the naso-nasal suture (state 2).

325. Texture of frontal bone. Some Paleogene hyracoids have frontal bones that are pitted and extremely rugose (state 1). All other known afrotherians have smooth frontal bones (state 0).

326. Palatine-frontal contact. Contains information similar to that of Novacek's [30] character 21. The palatine either does (state 1) or does not (state 0) contact the frontal bone within the orbital mosaic.

327. Palatine contribution to the orbital mosaic. Contains information similar to that of Asher's [2] character 25, Novacek's [30] character 21, and Court's [20] character 5. Afrotherians differ in the extent to which the palatine contributes to the orbital mosaic. Although a reduction in the size of the palatine often leads to a loss of contact between the frontal and the palatine, this character is not dependent on that of character 316 (palatine-frontal contact) because Amblysomus has a very small palatine bone (state 0) that nevertheless contacts the frontal within the orbital mosaic [32]. Other taxa have a palatine that expands into the orbital mosaic, in some cases contacting the lacrimal (state 1).

328. Palatal fenestration (MSO). Contains information similar to that of Novacek's [30] character 15. Macroscelidines exhibit a variable number of fenestrations in the palate, varying from one major fenestra (state 1) to multiple well-developed fenestrae (state 2). Novacek [30] has suggested that these foramina represent enlarged anterior and middle palatine foramina. In most taxa these enlarged fenestrations are absent (state 0).

329. Foramen rotundum (MSO). Court's [20] character 19, Novacek's [30] character 33, and MacPhee's [23] character 7. Placental mammals vary in the number of exit foramina transmitting the ophthalmic (V1) and maxillary (V2) branches of the trigeminal nerve. These two branches commonly travel together through a single sphenorbital fissure (state 2), but in some taxa an additional foramen for V2 (the foramen rotundum) is isolated from the foramen for V1 (the primitive sphenorbital fissure) by a bar of bone (state 0). Polymorphic expressions of states 0 and 2 are scored as state 1.

330. Optic foramen size (MSO). Contains information similar to that of Asher's [2] character 18, Novacek's [30] character 30, and MacPhee's [23] character 1). Some ingroup taxa have lost the optic foramen, the bony canal in the orbitosphenoid that transmits the optic nerve and associated vessels (state 2). Among those taxa that retain an optic foramen, the canal is either much smaller than the sphenorbital fissure (foramen for V1 or V1+V2) (state 1), or it is approximately the same size as the sphenorbital fissure (state 0).

331. Sinus canal. Contains information similar to that of Asher's [2] character 20 and Novacek's [30] character 38. When present, the sinus canal travels along the inner wall of the braincase, transmitting the stapedial ramus superior and/or a companion cranio-orbital sinus from the region of the middle ear into the orbitotemporal region (through a foramen that is itself often called the sinus canal or "cranio-orbital foramen"). The canal either enters the orbitotemporal region via a distinct foramen anterior, or just lateral to, the sphenorbital fissure and posterior to the ethmoid foramen (state 1), or the canal is confluent with the ethmoid foramen (state 2), or it is confluent with the sphenorbital fissure (state 3). Taxa without a sinus canal are scored as state 0.

332. Disposition of orbitotemporal foramina (excluding ethmoid foramen). Contains information similar to that of Court's [21] character 8. The orbitotemporal foramina (optic foramen, sphenorbital fissure, and, variably, foramen rotundum) are either posteriorly crowded in the rear of the orbit and are not clearly visible in lateral view (state 0), or they are spread out rostrocaudally so that one or more of the foramina are clearly visible in lateral view (state 1).

333. Crista orbitotemporalis (MSO). Some afrotherians have a crest of bone on the alisphenoid (and sometimes the frontal) in the orbitotemporal region -- here referred to as a "crista orbitotemporalis" -- that either encloses the orbitotemporal foramina or shields these foramina dorsally, often as a rostral extension of the bone that encloses the sphenorbital fissure laterally. In some taxa the crest extends only a short way beyond these foramina (state 2), but in derived proboscideans this crest is rostrally extensive (state 4), in some cases reaching the postorbital process. Most other taxa have no such crest (state 0). Polymorphic expressions of states 0 and 2 are scored as state 1; polymorphic expressions of states 2 and 4 are scored as state 3.

334. Suboptic foramen (Novacek's [30] character 31). Crown macroscelideans have two foramina positioned just ventral to the optic foramina -- here referred to as "suboptic" foramina (state 1). All other ingroup taxa lack these foramina (state 0).

335. Frontal-alisphenoid contact (MSO). Contains information similar to that of Court's [33] character 13 and Novacek's [30] character 29. A number of afrotherians lack any contact between the frontal and the alisphenoid within the orbitotemporal region (state 0). Among those taxa in which contact occurs, there are forms that exhibit processes from one or both bones that lead to only a very slight contact (state 2), while other taxa exhibit a relatively extensive contact of the frontal and the alisphenoid (state 4). Polymorphic expression of states 0 and 2 is scored as state 1; polymorphic expression of states 2 and 4 is scored as state 3.

336. Alisphenoid canal (MSO). Contains information similar to that of Asher's [2] character 10, Novacek's [30] character 34, Domning's [4] character 101, and Court's [20] character 20. The alisphenoid canal is generally situated just medial to foramen ovale and serves as a conduit for the maxillary artery (state 0). In many ingroup taxa this canal is absent (state 2). Polymorphic expressions of states 0 and 2 are scored as state 1.

337. Position of foramen ovale. Contains information similar to that of Domning's [4] character 103. Ingroup taxa vary in the extent to which the foramen ovale (the exit foramen for the mandibular division of the trigeminal nerve) is enclosed by bone. In most placentals the foramen ovale is completely enclosed within the alisphenoid (state 0), but in various afrotherians and other placentals the "foramen" forms nothing more than a notch or incisure in the caudal wall of the alisphenoid (state 1), along the rostral border of the piriform fenestra or middle ear cavity. 

338. Configuration of pterygoid region. Asher's [2] character 14 and Novacek's [30] character 36. Some ingroup taxa have pterygoid processes that form single laminae on each side of the nasal choana (state 0), and there is no clear development of a pterygoid fossa. In other taxa there are two processes on each side of the nasal choana (state 1). Some paenungulates have massive, ventrally projecting pterygoid processes (state 2). 

339. Crista galli (Asher's [2] character 35 and Novacek's [30] character 5). The crista galli is a plate of bone that projects dorsally along the intracranial exposure of the cribriform plate. It is either present (state 0) or absent (state 1).

340. Position of ethmoidal foramen. Contains information similar to that of Asher's [2] character 19, Court's [20] character 16. Among ingroup taxa that have an ethmoid foramen (which serves as a conduit for the arterial and venous blood supply and ethmoidal nerves to and from the ethmoid region), the foramen is placed either well rostral to the sphenorbital fissure (state 0), opens within the superior part of the sphenorbital fissure (state 1), or opens just above the optic foramen (state 2). Taxa that lack an ethmoid foramen are assigned state 3. 

341. Postorbital processes (MSO). Contains information similar to that of Domning's [4] character 36 and Novacek's [30] character 26. Among ingroup taxa the orbit can either be enclosed by a dorsal postorbital process or apophysis, but not by a ventral postorbital process (state 2), or by distinct dorsal and ventral postorbital processes (state 4). In some taxa these two processes join to form a complete postorbital bar (state 6). Many taxa lack postorbital processes (state 0). Polymorphic expression of states 0 and 2 is scored as state 1; polymorphic expression of states 2 and 4 is scored as state 3; polymorphic expression of states 4 and 6 is scored as state 5.

342. Sagittal crest (MSO). Contains information similar to that of Domning's [4] character 51. Among afrotherians the bilateral insertion sites for the temporalis musculature are either poorly developed or do not meet in the sagittal plane (state 0), meet at the very back of the cranium, forming a short sagittal crest (state 2), or meet far forward on the cranial roof, forming an elongate sagittal crest (state 4). Polymorphic expression of states 0 and 2 is scored as state 1; polymorphic expression of states 2 and 4 is scored as state 3.

343. Zygomatic arch. Contains information similar to that of Asher's [2] character 26, Novacek's [30] character 25, and MacPhee's [23] character 32. Most placentals have a rostrally directed process of the squamosal that contacts a caudally projecting process of the jugal, forming a complete zygomatic arch (state 0). Among afrotherians, tenrecs are unique in lacking such a connection, leaving a laterally exposed temporal fossa (state 1).

344. Most posterior point of maxillary jugal process relative to maxillary tooth row (MSO). Among afrotherians, the posterior aspect of the root of the maxillary jugal process is placed either posterior to M3 (state 0), along the length of M3 (state 2), along the length of M2 (state 4), along the length of M1 (state 6), or along the length of (or anterior to) P4 (state 8). Polymorphic expression of states 0 and 2 is scored as state 1; polymorphic expression of states 2 and 4 is scored as state 3; polymorphic expression of states 4 and 6 is scored as state 5; polymorphic expression of states 6 and 8 is scored as state 7.

345. Flaring of zygomatic arch (MSO). The zygomatic arch is either oriented rostrocaudally, with no lateral flare caudally (state 0), or the arch flares markedly towards its caudal termination (state 2). Polymorphic expression of states 0 and 2 is scored as state 1.

346. Robusticity of midpoint along zygomatic arch. Among ingroup taxa the zygomatic arch is either dorsoventrally slender, being shallower than, or approximately as dorsoventrally deep as, the mesiodistal length of M2 (state 0), or the arch is dorsoventrally deeper than the maximum mesiodistal width of M2 (state 1).

347. Squamosal contribution to zygomatic arch (MSO). Some paenungulates have a massive, dorsoventrally thick squamosal component in the zygomatic arch that either  tapers along its length to meet the jugal in a horizontal suture (state 1) or terminates in a broad convexity that approaches the orbit (state 2). Most afrotherians lack a dorsoventrally thick squamosal component in the zygomatic arch (state 0).

348. Caudal extension of jugal/contribution to glenoid fossa (MSO). Court's [20] character 12. A number of paenungulates have an expanded jugal component in the zygomatic arch that extends caudally to the point of the glenoid fossa (state 2); in some taxa the jugal actually contributes to the articular surface for the mandibular condyle (state 4). In many other afrotherians the jugal component of the zygomatic arch is small and does not reach the rostral border of the glenoid fossa (state 0). Polymorphic expression of states 0 and 2 is scored as state 1; polymorphic expression of states 2 and 4 is scored as state 3.

349. Extensive pneumatization of the cerebral aspect of the squamosal (Shoshani's [34] character 5). Most proboscideans have a squamosal that is greatly pneumatized dorsal to the glenoid fossa (state 1). Most other placentals lack this pneumatization (state 0).

350. Squamosal sinus canals (Novacek's [30] character 38). A number of ingroup taxa have discrete foramina along the squamosal-parietal suture dorsal to the region of the external auditory meatus (state 1). In at least some taxa this foramen presumably serves as an exit for the ramus temporalis of the stapedial ramus inferior [35], but canals in the same position have been interpreted as conduits for the superficial temporal vein in proboscideans [20]. Many afrotherians lack such foramina (state 0), which are here referred to as "squamosal sinus canals".

351. Postglenoid process. Contains information similar to that of Novacek's [30] character 39. Among afrotherians the postglenoid process, which is situated at the caudal end of the glenoid fossa for articulation with the mandibular condyle, can either be absent or very small (state 0), a small lip or lump of pneumatized bone (state 1), a dorsoventrally tall process (state 2), or recurved (state 3).

352. Postglenoid foramen (Court's [20] character 21, Novacek's [30] character 45). Many placentals have a foramen just caudal to the glenoid fossa that serves as a conduit for the capsuloparietal emissary vein (state 0). Among afrotherians, Orycteropus and paenungulates lack such a conduit (state 1).

353. Tympanic process of basisphenoid. Contains information similar to that of Asher's [2] character 1, MacPhee et al.'s [36] character 3, and Novacek's [30] character 49. Most placentals lack any contribution, or have only an insignificant contribution, of the basisphenoid to the auditory bulla (state 0). In some afrotherians the basisphenoid sends off a large tympanic process that provides a significant rostral wall to the middle ear cavity (state 1).

354. Basioccipital keel (MSO). Some afrotherians have a well-developed midline keel along the ventral surface of the basioccipital (state 4). In other taxa a keel is present but is poorly defined (state 2); many other taxa lack a keel (state 0). Polymorphic expression of states 0 and 2 is scored as state 1; polymorphic expression of states 2 and 4 is scored as state 3.

355. Basisphenoid pit (Asher's [2] character 11, Frost et al.'s [1] character 30). All tenrecines (with the exception of Hemicentetes) have a deep pit in the basisphenoid just rostral to the region of the middle ear cavity (state 1). Other afrotherians considered in this study lack such a "basisphenoid pit" (state 0).

356. Petrosal-basioccipital contact. It is common for placentals to have some sort of contact or fusion of the petrosal, some epitympanic wing of the petrosal, or of some bullar element, to the adjacent basioccipital (state 1). In some afrotherians the piriform fenestra is caudally continuous with a large space that isolates the petrosal and, when present, its bullar elements, from the basioccipital (state 0).

357. Morphology of tympanic roof. Contains information similar to Asher's [2] character 5, MacPhee et al.'s [36] character 8, and Frost et al.'s [1] character 33. Some afrotherians lack a bony roof rostral to the petrosal, leaving a large piriform fenestra for the entrance of the internal carotid artery into the braincase (state 2); other taxa have some development of a tympanic roof that is composed of the squamosal, alisphenoid, or both (state 0). Polymorphic expression of states 0 and 2 is scored as state 1.

358. Epitympanic sinus. Contains information similar to Asher's [2] character 6, MacPhee et al.'s [36] character 10, MacPhee's [23] character 14, and Frost et al.'s [1] character 39. Epitympanic sinuses are large vacuities that develop lateral to the epitympanic recess. Among the taxa examined only chrysochlorids and Orycteropus have been scored as having such a sinus (state 1), although these dilations may not be homologous because chrysochlorids' sinuses contain ear ossicles while Orycteropus' do not.

359. Perilymphatic foramen (Court's [20] character 28). In crown therians and some stem therians (e.g. Vincelestes), the primitive perilymphatic foramen or duct observable in more basal living and extinct mammals [e.g., 37, 38] becomes separated by the processus recessus, leading to the formation of a discrete fenestra cochleae and aqueductus cochleae [39] (state 0). In some fossil paenungulates, such as Arsinoitherium [20] and Moeritherium [40], as well as extant sirenians and proboscideans [41], the processus recessus fails to develop, leaving a large undivided perilymphatic foramen and no true fenestra cochleae (state 1). This feature has been cited as a synapomorphy of Tethytheria [41], but is now known to be absent in basal members of the proboscidean (Numidotherium, Phosphatherium) and sirenian (Prorastomus) clades [5, 40].

360. Prootic canal. The prootic canal serves as a conduit for the prootic sinus to enter the middle ear and join the lateral head vein, and occurs in monotremes, multituberculates, stem therians such as Vincelestes, some but not all [42] marsupials, and the stem placental Prokennalestes [38, 43] (state 0). No crown placentals are known to have a prootic canal (state 1).

361. Position of tympanic aperture for the facial nerve (Court's [20] character 29). After coursing through the internal acoustic meatus, cranial nerve VII enters into the tympanic cavity via the facial foramen either far rostral to the fenestra vestibuli (state 0), or lateral or posterior to, this fenestra (state 1).

362. Ectotympanic position/morphology. Contains information from Asher's [2] character 8, MacPhee et al.'s [36] character 5, and Novacek's [30] characters 47 and 48. The ectotympanic can either be aphaneric (obscured from ventral view by some component of the auditory bulla) (state 0), semiphaneric (partially obscured from ventral view) (state 1); phaneric but ring-like and unexpanded, providing no contribution to the auditory bulla (state 2); or laterally tubular and at least semiphaneric, expanding medially to enclose the lateral aspect of the bulla (state 3).

363. Alisphenoid contribution to the auditory bulla. The alisphenoid of Didelphis and extant macroscelideans provides a substantial component to the rostral wall of the auditory bulla (state 1). All other therians considered herein lack an alisphenoid component in the auditory bulla (state 0).

364. Entotympanic (MSO). Contains information similar to that of Novacek's [30] character 49. Entotympanics are ossifications that develop independently within the fibrous membrane of the tympanic cavity [44], and have a widespread distribution among placental mammals [e.g., 32, 45]. In this study entotympanic elements are scored as being either absent (state 0), present but providing an insignificant contribution to the bulla (state 1), or present and providing a substantial contribution to the bulla (state 2).

365. Subarcuate fossa (MSO). Court's [20] character 25, Novacek's [30] character 59, and MacPhee's [23] character 15. The subarcuate fossa of the petrosal encloses the parafloccular lobe of the cerebellum and varies from being present and very deeply excavated (state 0), to being present but poorly excavated (state 1), to being absent (state 2).

366. Caudal tympanic process of the petrosal. Contains information similar to that of Asher's [2] character 3, MacPhee et al.'s [36] character 2, MacPhee's [23] character 10, and Novacek's [30] character 2. Caudal tympanic processes of the petrosal can take many forms and are often difficult to categorize consistently into discrete character states [23]; in this study I have chosen to employ a scoring system modified from that of Asher [2] in which taxa with large caudal tympanic processes that "shield the fenestra cochleae, the entrance of the internal carotid artery as it enters the middle ear, and/or contribute to the posterior wall of the ossified auditory bulla" (p. 242) are assigned state 1, while those taxa in which the caudal tympanic process is small or only shields the fenestra cochleae ventrally are assigned state 0.

367. Rostral tympanic process of the petrosal (MSO). Asher's [2] character 4. Taxa with a rostral tympanic process that extends up to the ventral apex of the promontory, contributes to the entrance for the internal carotid artery, and/or articulates with the basisphenoid, are scored as state 0; taxa without such a process are assigned state 2. Polymorphic expression of states 0 and 2 is scored as state 1.

368. Arterial canals. Contains information similar to that of Novacek's [30] character 53 and Court's [20] character 32. Some afrotherians have bony canals that enclose one or more branches of the internal carotid artery as it passes across the promontorium or distal to the stapes (state 1); most placentals lack arterial canals (state 0).

369. Mastoid foramen. Among afrotherians mastoid foramina are either present (state 2) or absent (state 0). Polymorphic expression of states 0 and 2 is scored as state 1.

370. Mastoid exposure. Contains information similar to that of Asher's [2] character 17, Novacek's [30] character 60, MacPhee's [23] character 12, and Court's [20] character 36. In most placentals the mastoid is exposed between the squamosal and exoccipital along the caudal and ventral sides of the cranium. This exposure is present either posterior (state 0) or anterior (state 1) to the nuchal muscle scars. In paenungulates, the squamosal contacts the exoccipital along the ventrolateral aspect of the cranium, obscuring the mastoid from view (state 3). Although the mastoid of sirenians has a considerable amount of extracranial exposure through the mastoid foramen, they are assigned state 3 because they exhibit ventral contact of the squamosal and exoccipital, obscuring the mastoid from ventrolateral view (see, e.g., Novacek & Wyss [46], fig. 6F). Some macroscelidines exhibit mastoid exposure but the mastoid is not clearly delimited rostrally or caudally by nuchal muscle scars (state 2).

371. Approximation of posttympanic and postglenoid processes. Contains information similar to that of Court's [20] character 23 and Novacek's [30] character 46. In most placentals the posttympanic and postglenoid processes of the squamosal are widely separated along the ventrolateral aspect of the cranium (state 0). In some paenungulates the two processes closely approximate each other ventral to the external auditory meatus (state 1), while in derived elephantiforms the two processes are actually confluent ventral to this meatus (state 2). In some placentals the two processes closely approximate each other dorsal to the external auditory meatus (state 3).

372. Bones enclosing the foramen magnum (Court's [20] character 39, Domning's [4] character 66). The foramen magnum is either enclosed by the supraoccipital dorsally and the exoccipital laterally (state 0), or entirely by the exoccipital (state 1).

373. Hypoglossal foramen (MSO). Contains information similar to that of Court's [20] character 38 and Asher's [2] character 12. Almost all placentals have small foramina in the occipital for passage of cranial nerve XII (state 1). Various paenungulates have lost a discrete hypoglossal foramen (state 0), while the tenrecid Potamogale has greatly enlarged hypoglossal foramina (state 2).

374. Paroccipital processes. Contains information similar to that of Court's [20] character 37 and Novacek's [30] character 65. The exoccipital of various taxa exhibits a "paroccipital process" for attachment of neck musculature that is well-developed and protrudes ventrally (state 1). Other taxa either lack paroccipital processes, or have only poorly-developed processes (state 0).

375. Morphology of nuchal process. In many afrotherians the nuchal muscle scars form low rugosities, and there is no development of caudally projecting processes (state 0). In other taxa the nuchal process is shelf-like and caudally projecting (state 2), while in some Paleogene hyracoids the nuchal process is caudally projecting and bifurcate (state 1).

376. Pneumatization of cranial vault. Derived elephantiforms have cranial bones that are extensively pneumatized with numerous large air cells (state 1). All other afrotherian taxa lack the level of pneumatization seen in these proboscideans (state 0).

377. Posterior stapedial ramus (Asher's [28] character 1). The posterior ramus of the stapedial artery is either reduced or absent (state 1) or present (state 0).

378. Superior stapedial ramus (Asher's [28] character 2 and Court's [20] character 34). The superior ramus of the stapedial artery is either present (state 0), or absent, with the supply of cranial structures such as eyes and meninges taken over by other arterial sources (state 1).

379. Inferior stapedial ramus (Asher's [28] character 3). The inferior stapedial ramus is either reduced or absent (state 1) or present (state 0).

380. Inferior stapedial foramen (Asher's [47] character 31). Among afrotherians there is either a discrete foramen for the inferior stapedial ramus formed by alisphenoid contribution to anterior wall of middle ear (state 1), or there are no distinct arterial foramina for the inferior stapedial ramus as it leaves middle ear (state 0).

381. Origin of ophthalmic artery (Asher's [28] character 4). The ophthalmic artery either arises from the internal carotid (state 0), the ramus superior of the stapedial artery (after passing through the sinus canal) (state 1), from a ramus infraorbitalis supplied by the ramus inferior of the stapedial artery (state 2), or from the external carotid artery (state 3).

382. Origin of ramus mandibularis (Asher's [28] character 5). The ramus mandibularis either originates from the inferior ramus of the stapedial artery (state 0) or the external carotid artery (state 1).

383. Location of internal carotid artery relative to the anterior pole of cochlea (Asher's [28] character 6). As the internal carotid artery enters the braincase it either passes medial (state 0) or lateral (state 1) to the anterior pole of the pars cochlearis of the petrosal.

384. Proximal course of ramus infraorbitalis (Asher's [28] character 8). The ramus infraorbitalis either takes an extracranial course (state 0) or it travels intracranially ventral to the trigeminal nerve within the cavum epiptericum (state 1).

385. Course of proximal external carotid artery (Asher's [28] character 9). The external carotid artery either passes medial (state 0) or lateral (state 1) to thyropharyngeus.

General

386. Pachyostosis. All known living and extinct sirenians exhibit pachyostosis in their skeletal anatomy (state 1). All other afrotherians lack this condition (state 0).

Reproductive/developmental characters

387. Scrotum. Among afrotherians the scrotum is either present (state 0) or absent (state 1).

388. Testicular descent (MSO). Character states are those discussed by Setchell [48]. Among placentals the testicles can either be completely undescended and situated just caudal to the kidneys (state 0), slightly descended from this position to be situated near the bladder (state 1), descended to, or just through, the ventral abdominal wall (state 2), descended into a cremasteric sac (state 3), descended into a non-pendulous scrotum without a distinct neck (state 4), or descended into a pendulous scrotum with a distinct neck (state 5). 

389. Gross form of the chorioallantoic placenta (Mess and Carter's [49] character 1). Under the assumption that the sampled ingroup taxa share the character states of their close relatives, for the following placental characters Amblysomus was assigned the states listed in Mess and Carter [49] for the chrysochlorid Eremitalpa; Potamogale was assigned the states listed for Micropotamogale; Dasypus was assigned the states listed for xenarthran Bradypus; and Condylura was assigned those states that overlapped in the talpids Scalopus and Talpa. Among ingroup taxa the chorioallantoic placenta is, near term, either diffuse (state 0), zonary (state 1), cotyledonary (state 2), discoid (state 3), or double discoid (state 4).

390. Interhemal barrier (Mess and Carter's [49] character 4). The interhemal barrier is either epitheliochorial (state 0), endotheliochorial (state 1), or hemochorial (state 2).

391. Type of trophoblast in barrier (Mess and Carter's [49] character 5). Either a cytotrophoblast (state 0), a syncytiotrophoblast (state 2), or both (state 1) are present in the barrier.

392. Hemophagous regions (Mess and Carter's [49] character 7). Hemophagous regions are either present (state 0) or absent (1).

393. Areolae (Mess and Carter's [49] character 8). Areolae are either present (state 0) or absent (state 1).

394. Differentiation of endometrial stroma (Mess and Carter's [49] character 9). Decidual cells are either present (state 1) or atypical or absent (state 0).

395. Implantation (first attachment) (Mess and Carter's [49] character 10). Implantation is either central (state 0), mesometrial (state 1), antimesometrial (state 2), or lateral (state 3).

396. Amniogenesis (Mess and Carter's [49] character 12). Amniogenesis is either by cavitation (state 0) or folding (state 1).

397. Allantoic vesicle chambers (Mess and Carter's [49] character 18). There are either one (state 0), four (state 1), two (state 2), or no (state 3) allantoic vesicle chambers.

398. Urogenital opening (Mess and Carter's [49] character 19). The urogenital opening is either separate (state 1) or a “cloaca” is present (state 0).

399. Type of uterus (Mess and Carter's [49] character 20). Among ingroup taxa the uterus is either duplex (state 0), bicornuate (state 1), or simplex (state 0).

400. Newborn (Mess and Carter's [49] character 21). Newborn are either precocial, with their eyes open at birth (state 0), or altricial, with their eyes closed at birth (state 1).


References:

1.	Frost DR, Wozencraft WC, Hoffman RS: Phylogenetic relationships of hedgehogs and gymnures (Mammalia: Insectivora: Erinaceidae). Smithson Contrib Zool 1991, 518:1–69.
2.	Asher RJ: A morphological basis for assessing the phylogeny of the "Tenrecoidea" (Mammalia, Lipotyphla). Cladistics 1999, 15:231-252.
3.	DeBlieux DD, Simons EL: Cranial and dental anatomy of Antilohyrax pectidens: a late Eocene hyracoid (Mammalia) from the Fayum, Egypt. J Vert Paleo 2002, 22:122-136.
4.	Domning DP: A phylogenetic analysis of the Sirenia. Proc San Diego Soc Nat Hist 1994, 29:177-189.
5.	Gheerbrant E, Sudre J, Tassy P, Amaghzaz M, Bouya B, Iarochene M: Nouvelles données sur Phosphatherium escuilliei (Mammalia, Proboscidea) de l'Éocène inférieur du Maroc, apports à la phylogénie des Proboscidea et des ongulés lophodontes. Geodiversitas 2005, 27(2):239-333.
6.	Domning DP, Hayek L-AC: Horizontal tooth replacement in the Amazonian manatee (Trichechus inunguis). Mammalia 1984, 48(1):105-127.
7.	MacInnes DG: Fossil Tubulidentata from East Africa. Fossil Mammals of Africa 1956, 10:1-52.
8.	Archibald JD, Averianov AO: Paranyctoides and allies from the late Cretaceous of North America and Asia. Acta Palaeontologica Polonica 2001, 46(4):533-551.
9.	Sigogneau-Russell D, Dashzeveg D, Russell DE: Further data on Prokennalestes (Mammalia, Eutheria inc. sed.) from the early Cretaceous of Mongolia. Zoologica Scripta 1992, 21(2):205-209.
10.	Ji Q, Luo Z-X, Yuan C-X, Wible JR, Zhang J-P, Georgi JA: The earliest known eutherian mammal. Nature 2002, 416:816-822.
11.	Nessov LA, Sigogneau-Russell D, Russell DE: A survey of Cretaceous tribosphenic mammals from middle Asia (Uzbekistan, Kazakhstan, and Tajikistan), of their geological setting, age and faunal environment. Palaeovertebrata 1994, 23:51-92.
12.	Nessov LA, Archibald JD, Kielan-Jaworowska Z: Ungulate-like mammals from the late Cretaceous of Uzbekistan and a phylogenetic analysis of Ungulatomorpha. Bull Carnegie Mus Nat Hist 1998, 34:40-88.
13.	Lillegraven JA: Latest Cretaceous mammals of the upper part of the Edmonton Formation of Alberta, Canada, and review of marsupial-placental dichotomy in mammalian evolution. University of Kansas Paleontological Contributions 1969, 50:1-122.
14.	Clemens WA: Fossil mammals of the Type Lance Formation, Wyoming. Part III. Eutheria and summary. University of California Publications in Geological Sciences 1973, 94:1-102.
15.	Novacek MJ: The primitive eutherian dental formula. J Vert Paleo 1986, 6(2):191-196.
16.	Cifelli RL: Counting premolars in early eutherian mammals. Acta Palaeontologica Polonica 2000, 45(2):195-198.
17.	Rougier GW, Wible JR, Novacek MJ: Implications of Deltatheridium for early marsupial history. Nature 1998, 396:459-463.
18.	Cifelli RL: Tribosphenic mammal from the North American Early Cretaceous. Nature 1999, 401:363-366.
19.	Rasmussen DT, Simons EL: New Oligocene hyracoids from Egypt. J Vert Paleo 1988, 8:67-83.
20.	Court N: The skull of Arsinoitherium (Mammalia, Embrithopoda) and the higher order interrelationships of ungulates. Palaeovertebrata 1992, 22(1):1-43.
21.	Court N: A new species of Numidotherium (Mammalia: Proboscidea) from the Eocene of Libya and the early phylogeny of the Proboscidea. J Vert Paleo 1995, 15(3):650-671.
22.	Andrews CW: A descriptive catalogue of the Tertiary Vertebrata of the Fayum, Egypt; based on the collection of the Egyptian Government in the Geological Museum, Cairo, and on the collection in the British Museum (Natural History): Trustees of the British Museum (Natural History); 1906.
23.	MacPhee RDE: Morphology, adaptations, and relationships of Plesiorycteropus, and a diagnosis of a new order of eutherian mammals. Bull Am Mus Nat Hist 1994, 220:1-214.
24.	Dobson GE: A monograph of the Insectivora, systematic and anatomical. London: Van Voorst; 1882-90.
25.	Puttick GM, Jarvis JUM: The functional anatomy of the neck and forelimbs of the Cape golden mole, Chrysochloris asiatica (Lipotyphla, Chrysochloridae). Zoologica Africana 1977, 12:445-458.
26.	Pickford M, Moyà-Solà S, Mein P: A revised phylogeny of Hyracoidea (Mammalia) based on new specimens of Pliohyracidae from Africa and Europe. N Jb Geol Paläont Abh 1997, 205:265-288.
27.	Novacek MJ: The skull of leptictid insectivorans and the higher-level classification of eutherian mammals. Bulletin of the American Museum of Natural History 1986, 183(1):1-111.
28.	Asher RJ: Cranial anatomy in tenrecid insectivorans: Character evolution across competing phylogenies. Am Mus Nov 2001, 3352:1-54.
29.	Sánchez-Villagra M: Ontogenetic and phylogenetic transformations of the vomeronasal complex and nasal floor elements in marsupial mammals. Zool J Linn Soc 2001, 131:459–479.
30.	Novacek MJ: The skull of leptictid insectivorans and the higher-level classification of eutherian mammals. Bull Am Mus Nat Hist 1986, 183:1-112.
31.	Whitworth T: The Miocene hyracoids of east Africa, with some observations on the order Hyracoidea. Fossil Mammals of Africa 1954, 7:1-58.
32.	MacPhee RDE, Novacek MJ: Definition and relationships of Lipotyphla. In: Mammal Phylogeny: Placentals. Edited by Szalay FS, Novacek MJ, McKenna MC, vol. 2. Berlin: Springer-Verlag; 1993: 13-31.
33.	Court N: An enigmatic new mammal from the Eocene of north Africa. J Vert Paleo 1992, 13:267-269.
34.	Shoshani J: Para- or monophyly of the gomphotheres and their position within Proboscidea. In: The Proboscidea: Evolution and Palaeoecology of Elephants and their Relatives. Edited by Shoshani J, Tassy P. Oxford: Oxford University Press; 1996: 149-177.
35.	Wible JR: Cranial circulation and relationships of the colugo Cynocephalus (Dermoptera, Mammalia). American Museum Novitates 1993, 3072:1-27.
36.	MacPhee RDE, Novacek MJ, Storch G: Basicranial morphology of early Tertiary erinaceomorphs and the origin of primates. American Museum Novitates 1988, 2921:1-42.
37.	Wible JR, Hopson JA: Basicranial evidence for early mammal phylogeny. In: Mammal Phylogeny: Mesozoic Differentiation, Multituberculates, Monotremes, Early Therians, and Marsupials. Edited by Szalay FS, Novacek MJ, McKenna MC. New York: Springer; 1993: 45-62.
38.	Wible JR, Rougier GW: Cranial anatomy of Kryptobaatar dashzevegi (Mammalia, Multituberculata) and its bearing on the evolution of mammalian characters. Bull Am Mus Nat Hist 2000, 247:1-124.
39.	Zeller U: Die Ontogenese und Morphologie der Fenestra rotunda und des Aquaeductus cochleae von Tupaia und anderen Säugern. Gegenbaurs Morphol Jahrb 1985, 131:179-204.
40.	Court N: The periotic of Moeritherium (Mammalia, Proboscidea): homology or homoplasy in the ear region of Tethytheria McKenna, 1975? Zoological Journal of the Linnaean Society 1994, 112:13-28.
41.	Fischer MS, Tassy P: The interrelations between Proboscidea, Sirenia, Hyracoidea, and Mesaxonia: the morphological evidence. In: Mammal Phylogeny: Placentals. Edited by Szalay FS, Novacek MJ, McKenna MC, vol. 2. New York: Springer Verlag; 1993: 217-243.
42.	Sánchez-Villagra M, Wible JR: Patterns of evolutionary transformation in the petrosal bone and some basicranial features in marsupial mammals, with special reference to didelphids. Journal of Zoological Systematics and Evolutionary Research 2002, 40:26-45.
43.	Wible JR, Rougier GW, Novacek MJ, McKenna MC: Earliest eutherian ear region: A petrosal referred to Prokennalestes from the early Cretaceous of Mongolia. Am Mus Nov 2001, 3322:1-44.
44.	MacPhee RDE: Auditory regions of primates and eutherian insectivores: Morphology, ontogeny, and character analysis. Contributions to Primatology 1981, 18:1-281.
45.	Gaudin TJ, Wible JR: The entotympanic of pangolins and the phylogeny of the Pholidota (Mammalia). J Mamm Evol 1999, 6:39-65.
46.	Novacek MJ, Wyss AR: Higher-level relationships of the Recent eutherian orders: Morphological evidence. Cladistics 1986, 2:257-287.
47.	Asher RJ: Phylogenetic history of tenrecs and other insectivoran mammals. Ph.D. dissertation. Stony Brook, New York: SUNY Stony Brook; 2000.
48.	Setchell BP: The Mammalian Testis. Ithaca, New York: Cornell University Press; 1978.
49.	Mess A, Carter AM: Evolutionary transformations of fetal membrane characters in Eutheria with special reference to Afrotheria. J Exp Zool (Mol Dev Evol) B 2006, 306:140–163.
